# Metabolic network segmentation: A probabilistic graphical modeling approach to identify the sites and sequential order of metabolic regulation from non-targeted metabolomics data

**DOI:** 10.1371/journal.pcbi.1005577

**Published:** 2017-06-09

**Authors:** Andreas Kuehne, Urs Mayr, Daniel C. Sévin, Manfred Claassen, Nicola Zamboni

**Affiliations:** 1 Institute of Molecular Systems Biology, ETH Zurich, Switzerland; 2 PhD Program Systems Biology, Life Science Zurich Graduate School, Zurich, Switzerland; Centre for Research and Technology-Hellas, GREECE

## Abstract

In recent years, the number of large-scale metabolomics studies on various cellular processes in different organisms has increased drastically. However, it remains a major challenge to perform a systematic identification of mechanistic regulatory events that mediate the observed changes in metabolite levels, due to complex interdependencies within metabolic networks. We present the metabolic network segmentation (MNS) algorithm, a probabilistic graphical modeling approach that enables genome-scale, automated prediction of regulated metabolic reactions from differential or serial metabolomics data. The algorithm sections the metabolic network into modules of metabolites with consistent changes. Metabolic reactions that connect different modules are the most likely sites of metabolic regulation. In contrast to most state-of-the-art methods, the MNS algorithm is independent of arbitrary pathway definitions, and its probabilistic nature facilitates assessments of noisy and incomplete measurements. With serial (i.e., time-resolved) data, the MNS algorithm also indicates the sequential order of metabolic regulation. We demonstrated the power and flexibility of the MNS algorithm with three, realistic case studies with bacterial and human cells. Thus, this approach enables the identification of mechanistic regulatory events from large-scale metabolomics data, and contributes to the understanding of metabolic processes and their interplay with cellular signaling and regulation processes.

## Author summary

Reciprocal crosstalk between metabolism and cellular signaling pathways plays a crucial role in cellular decision-making. In recent years, this premise has motivated several metabolomics studies that aimed to gain a mechanistic understanding of metabolic phenotypes. However, due to complex interactions within metabolic networks, it remains a challenge to infer mechanisms that underlie metabolome changes. We present the metabolic network segmentation approach, a novel method that aimed to identify the sites and sequential order of metabolic regulatory events, based merely on steady state or dynamic metabolomics data. This method employs probabilistic graphical models to partition the entire metabolic network into modules with correlated metabolites. It identifies fractures between modules (i.e., reactions that connect non-correlated metabolites) as sites of regulation. We performed validation and benchmark analyses in hundreds of *E*. *coli* knockout mutants deficient in enzymes and transcription factors. Moreover, we verified the capability of our method for identifying the sequential order of metabolic regulatory events by testing it on fibroblasts exposed to oxidative stress. Our metabolic network segmentation algorithm is widely applicable, and thus, it will enhance our mechanistic understanding of metabolic phenotypes and their connections to cellular signaling and regulatory processes.

This is a PLoS Computational Biology Methods paper.

## Introduction

The consolidated notion that metabolites can provide feedback to cellular signaling and alter metabolism is a hallmark of several diseases. This notion has boosted interest in gaining a mechanistic and quantitative understanding of metabolic phenotypes [1–3]. Consequently, researchers in the field of metabolomics have established a battery of methods for large-scale analyses of metabolite levels in all sample types [4]. Continual advances in instrumentation and protocols for metabolomics have led to an exponential production of high-quality data on the metabolome. The quality of the data is reflected in the high number of detectable metabolites and the high experimental reproducibility. Due to these advances, it has become common that every study discovers multiple significant metabolite changes. This positive trend, however, has exacerbated the problem of interpreting metabolome changes.

In the context of systems or cellular biology, interpretations of metabolome data aim to generate testable hypotheses about the molecular events that might have led to the observed metabolome pattern. The quest for mechanisms underlying metabolome changes is complicated by the complex relationship between metabolite levels, enzyme properties, and metabolic fluxes [5, 6]; by the dozens of pathways connected to key metabolites; and by our partial understanding of cellular regulation [7]. This complexity is a non-trivial problem that cannot be addressed with traditional uni- and multivariate statistical techniques, which are generally used to identify markers or classify samples [7]. Instead, prior knowledge of the metabolic or regulatory networks must be embedded into the analysis for an efficient inference of the links that gave rise to the observed metabolome changes.

Mechanistic model-based approaches enable to model the dynamics of the metabolic network and its interactions at molecular level. Therefore these modelling approaches have been commonly considered the methods of choice for inferring mechanisms and regulatory events [8]. However, such an in depth mechanistic description of metabolism requires detailed knowledge of the model structure and the kinetic parameters, which in many cases limits model size and applicability (reviewed in [8]). Recent ensemble modelling approaches employed high dimensional fluxomics datasets to generate large-scale kinetic metabolic models of the well-studied model organism *Escherichia coli*. These approaches have been successful in, for example, estimating flux changes and metabolite yields metabolism of *Escherichia coli*, predicting metabolite yields in engineered *E*. *coli* enzyme mutant *strains* [9, 10]. Yet in many studies only metabolite levels and not metabolic fluxes are available. Therefore mechanistic modelling approaches for exclusively metabolomics data have been limited to well-defined, small metabolic models with known reaction stoichiometry and only a few dozen reactions and metabolites. Nevertheless, these models were highly successfully applied for example to identify regulations from absolutely quantified metabolomics data and dynamic modeling [11–13]. However, modern, large-scale metabolomics tools enable one to profile hundreds to thousands of metabolites throughout the metabolic network [14]. For such large metabolomics datasets, in most cases only less well-defined models with at least ten-fold more features, reactions, and parameters are available; thus, current mechanistic-based approaches are prone to failure.

Consequently, an ongoing effort to develop alternative, simpler computational tools has capitalized on current knowledge of metabolic networks to facilitate detailed interpretations of large-scale metabolomics data. These tools include metabolic pathway enrichment analyses and visualizations of metabolite changes on maps of metabolic networks (Reviewed in [7]). These approaches can simplify the analysis and interpretation of metabolomics data, but they can also be limited by their reliance on fixed significance cutoffs for grouping metabolites, fixed pathway definitions, and highly user-biased interpretations. These limitations can preclude discovery of unexpected regulatory events. Two approaches, namely reporter reactions and mass action ratios, were developed to make automatic predictions of regulatory sites, based on metabolomics data [15, 16]. Both these approaches are based on the assumption that a given metabolic perturbation, such as inhibiting a metabolic enzyme, will induce the strongest, most significant metabolic alterations in the levels of substrates and products. To date, these approaches have been solely applied to small metabolic subnetworks with a few dozen metabolites, of which almost all were measured. However, these methods tend to give misleading results in regions that are sparsely covered in an analytical metabolome, like peripheral pathways. Thus, randomly sampled data would have to replace undetected metabolites, and presumably, model performance would decline [16].

**Fig 1:**
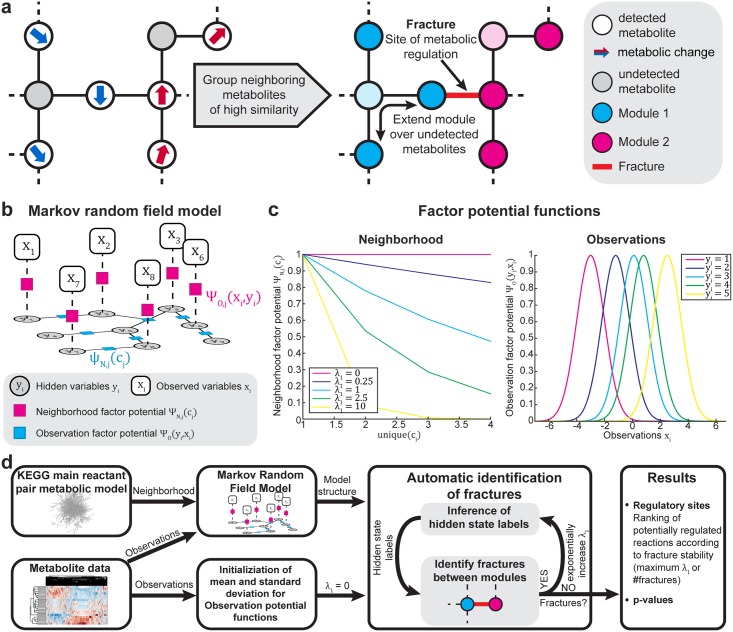
Design and implementation of the metabolic network segmentation algorithm, a probabilistic graphical modelling approach to identify sites of metabolic regulation. (a) Principle of the algorithm to identify sites of metabolic regulation using metabolomics data and metabolic network reconstructions. The algorithm segments the metabolic network into modules of highly similar changes. To deal with sparse and noisy metabolite measurements, it considers dependencies between neighboring metabolites to extend the modules. Fractures between two modules are potential sites of metabolic regulation. (b) Structure of the Markov random field model. The discrete hidden variables *y* represent the modules to which a metabolite belongs, the observed variables *x* integrate the metabolite measurements. The factor potential functions introduce probabilistic dependencies between neighboring hidden states (*ψ*_*N*_) and between measurements and the hidden states (*ψ*_*O*_). (c) Example of neighborhood and observation factor potential functions. The neighborhood factor potentials are module label dependent exponential decay functions. The influence of the metabolic neighborhood is controlled by *λ*_1_. Observation potential functions link hidden state/module labels to metabolic observations by hidden state dependent Gaussian distributions. (d) Algorithm workflow of the scan mode implementation to automatically identify sites of metabolic regulation. Details in S1 Fig.

To overcome this issue, we developed the metabolic network segmentation (MNS) algorithm. This generalized approach aimed to identify the regulated metabolic reactions responsible for the end point or dynamics of large-scale metabolomics experiments. In contrast to other approaches, which aim to reconstruct networks based on correlations between metabolites [17–19], we sought to identify reactions in the metabolic network that exhibited broken symmetry; i.e., metabolic changes, where substrates and products were not correlated. Our algorithm partitioned the metabolic network into regions of neighboring metabolites with consistent changes, and it identified reactions between two regions—so called fractures—as potential sites of metabolic regulation (Fig 1a).

Our algorithm is based on the undirected subclass of probabilistic graphical models, known as Markov random fields (MRFs), which were introduced by Lenz and Ising in the 1920’s to describe ferromagnetic materials [20]. MRFs consist of nodes that represent random variables and arcs that describe the probabilistic dependencies between connected random variables [21]. Therefore, MRFs have been successfully applied in diverse machine learning tasks that include noisy and incomplete data with underlying sequential or spatial structure [21, 22]. For example, in computer vision, these models are used for image segmentation or image reconstruction, where they employ dependency assumptions between neighboring pixels [22]. Likewise, the metabolic network defines an underlying spatial structure, where the substrates and products of a reaction are dependent. For example, given an unperturbed reaction, one would assume that changes in substrate and product levels should correlate. Given the structured nature of metabolism, MRFs are an ideal approach for pattern recognition in biochemical networks.

Here, we demonstrate that our approach outperformed current state-of-the-art algorithms in the identification of known regulatory sites and the prediction of novel regulatory sites. Moreover, we extended the algorithm for metabolomics data with serial structure, such as time courses. This unique extension enabled the model both to identify sites and also to determine the sequential order of events, i.e., the timing of metabolic regulatory steps.

## Design and implementation of the MNS algorithm

### Inference of metabolic regulation sites from univariate metabolomics data

The goal of our approach was to predict sites of metabolic regulation, such as activated or inhibited enzymes, from differential metabolomics data. These data were obtained from a univariate comparison of metabolite levels across conditions. Supported by experimental data [15, 16], we designed the MNS algorithm based on the assumption that the strongest metabolic changes occur in close proximity of a perturbed enzyme (Fig 1a). For example, when a flux-carrying enzyme is inhibited by a drug, we expected that the substrate and its precursors would accumulate, and the product levels would decrease. In contrast to existing methods, we did not focus solely on the direct substrate and products for the inference; instead, we aimed to segment the entire metabolic network into regions of consistent metabolite changes, so-called metabolic modules, where fractures between two modules are potential sites of metabolic regulation (Fig 1a). To deal with sparse and noisy metabolite measurements, our algorithm considered interdependencies between neighboring metabolites for predictions; i.e., we assumed that reactant levels of an unperturbed reaction would correlate.

Our algorithm was designed to build on organism-specific, genome-wide, metabolic network reconstructions. It employs main reactant-pair models, obtained from the KEGG database with a modified version of the MetaboNetworks toolbox [23, 24]. For each metabolite in the network, we introduced a discrete hidden variable, y_i_ ∈ **y**, into a MRF model (Fig 1b). The discrete values of the hidden variables represented the labels of the metabolic modules to which a certain metabolite belonged. Specifically, metabolites with similarly decreasing, increasing, or constant metabolite levels were likely to have the same label. The module labels of the hidden variables were not known *a priori* (hidden), but they were inferred by the algorithm through optimization of the conditional likelihood.

To account for network structure and proximity, we defined probabilistic dependencies between hidden variables that represented the reactants of the main reactant pairs (Fig 1b). Assuming that, in an unperturbed system, neighboring metabolites should be in the same module, we enforced a local homogeneity between neighboring hidden variables by introducing for each maximal clique (c_j_) in the hidden layer, a neighborhood factor potential, ψ_N,j_(c_j_). The maximal clique was defined as a maximal subset of nodes (i.e., metabolites), which were all connected to each other. The neighborhood factor potential ψ_N,j_(c_j_) was a hidden state, label dependent, exponential decay function, defined by:

$$\psi_{N,j}\left(c_{j}\right)=exp\left(-\lambda_{1}\cdot \left(\frac{unique\left(c_{j}\right)-1}{size\left(c_{j}\right)}\right)\right),$$

where the *unique(c*_*j*_*)* equals the number of different labels of hidden variables involved in a maximal clique, *c*_*j*_; the *size*(*c*_*j*_) is the number of hidden variables in the *c*_*j*_; and *λ*_*1*_ is a weighting factor that controls the strength of the neighborhood influence (Fig 1c).

Because the states of the hidden variables were required to be dependent on the metabolite data, for each detected metabolite of a given dataset, we introduced a continuous observed variable, x_i_ ∈ **x**, which represented the metabolic change, based on a univariate comparison, e.g., the log_2_(fold-change) or the z-scores (Fig 1b). The dependency between hidden and observed states was described by an observation factor potential, ψ_O,i_(x_i_,y_i_), which described their relationship with hidden state-dependent Gaussian distributions, as follows

$$\psi_{O,i}\left(x_{i},y_{i}\right)=exp\left(-\frac{{\left(x_{i}-\mu \left(y_{i}\right)\right)}^{2}}{2\sigma {\left(y_{i}\right)}^{2}}\right),$$

where *μ(y*_*i*_*)* and *σ(y*_*i*_*)* are the hidden state-dependent mean values and standard deviations, respectively (Fig 1c). The hidden state-dependent mean values were defined as either the mean values of the clusters obtained from a k-means clustering (k-means) analysis, or as the values equally distributed between the 0.001 and 0.999 quantiles of the complete dataset (quantile, S1 Table). The standard deviations can be set to a constant user-defined value (fixed); or they can be individually obtained from the standard deviations of the k-means clusters (k-means); or they can be set to the standard deviation of the complete dataset (all data, S1 Table). Together, the neighborhood and observation factor potentials of all metabolites, i ∈ M, and all maximal cliques, j ∈ C, describe the conditional likelihood of the MRF model, defined as

$$p\left(y|x\right)=\frac{1}{Z\left(x\right)}\overset{C}{\underset{j=1}{\prod }}exp\left(-\lambda_{1}\cdot \left(\frac{unique\left(c_{j}\right)-1}{size\left(c_{j}\right)}\right)\right)\overset{M}{\underset{i=1}{\prod }}exp\left(-\frac{{\left(x_{i}-\mu \left(y_{i}\right)\right)}^{2}}{2\sigma {\left(y_{i}\right)}^{2}}\right).$$

By maximizing the conditional likelihood, the optimal hidden state label distribution, $\hat{y}$, can now be determined as follows:

$$\hat{y}=\underset{y}{\text{argmax}}p\left(y|x\right).$$

Due to the complexity of our network, we employed an approximation algorithm called LazyFlipper, available in the OpenGM toolbox, to infer the best hidden states [25, 26].

To infer regulatory sites, our algorithm repetitively segmented the metabolic network by estimating the most likely hidden states, $\hat{y}$, with increasing neighborhood influence, λ_1_ (S1 Fig). The fractures between individual modules represented potential regulatory sites. This procedure was inspired by the watershed algorithm for image segmentation [27]; it assumes that the fractures identified at a high neighborhood influence, λ_1_, are most likely biologically meaningful. Sequential scanning through λ_1_ values was performed in a two-step process. In the initial step, λ_1_ was exponentially increased until all metabolites were assigned to the same module, and no more fractures were found. Then, in the second step, an extensive search within a linear range of λ_1_ was performed, which resulted in a list of fractures for each given λ_1_. The likelihood that each fracture represented a site of metabolic regulation was quantified with two entities. One entity was the fracture counts (#fractures), i.e., for how many values of λ_1_ can a given reaction be identified as a fracture between two modules? The second entity was the maximum λ_1_ value (max(λ_1_)) at which a reaction remained classified as a fracture (Fig 1d, S1 and S2 Figs). The significance of the fractures could then be determined with a permutation test, by comparing the real outcome with the outcome from 1000 repetitions of the analysis performed with permuted metabolite labels (S1 Fig). The p-value for each reaction, with max(λ_1_) = λ* and #fractures = n, can then be calculated as follows:

$$p_{max\left(\lambda_{1}\right)}\left(\lambda^{\text{*}}\right)=\frac{\#permutations\left(max\left(\lambda_{1}\right)\geq \lambda^{\text{*}}\right)}{\#permutations}$$

and

$$p_{\#fractures}\left(n\right)=\frac{\#permutations\left(\#fractures\geq n\right)}{\#permutations}.$$

To combine the inference results of individual predictors with different parameterizations, the max(λ_1_) and #fracture rankings were integrated with the rank product. The most likely regulated reactions were ordered by ascending rank products. Significance for the rank products was calculated, as described previously [28].

### Identification of the sequential order of metabolic regulation steps from dynamic metabolomics data

**Fig 2:**
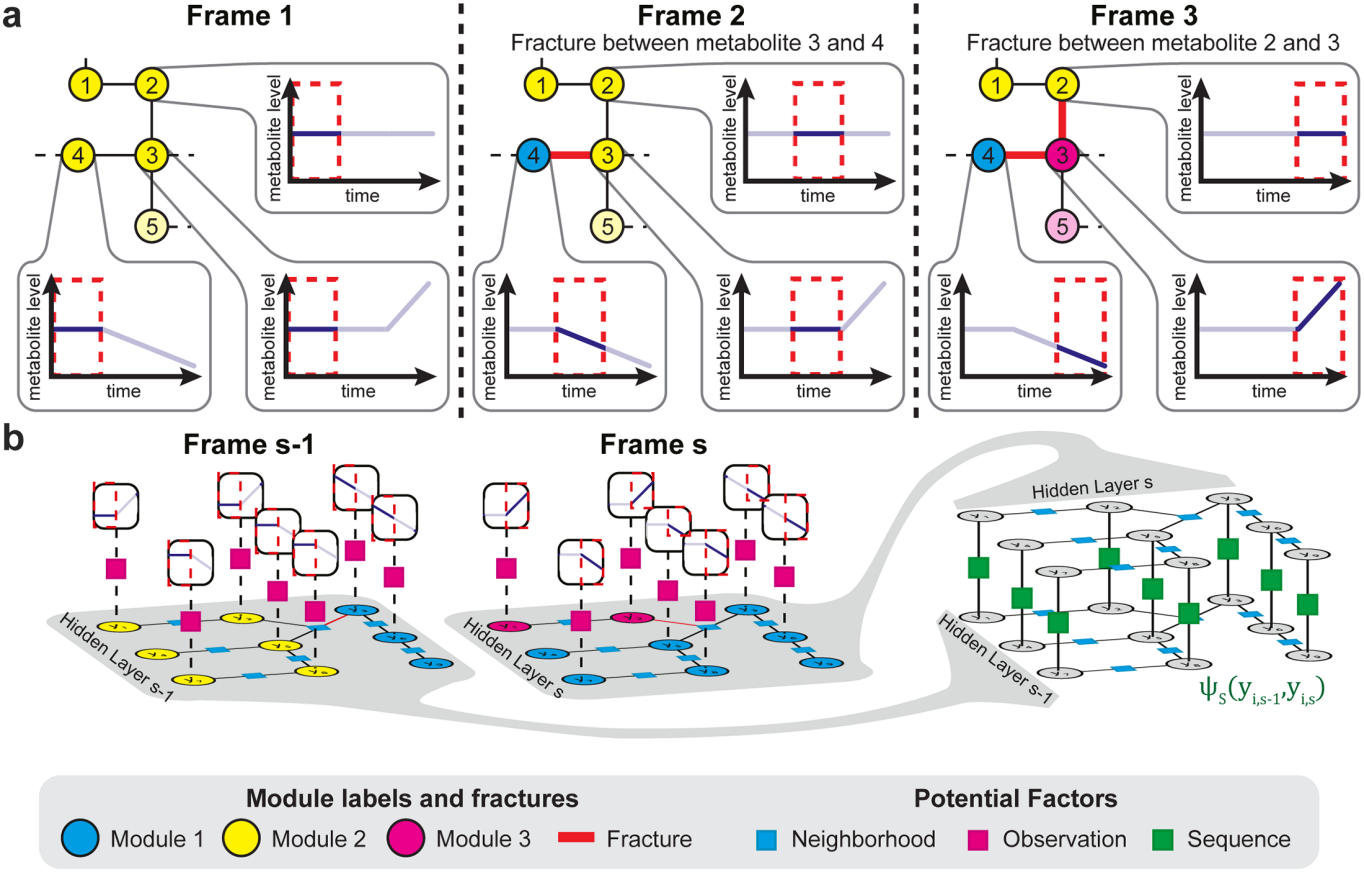
Principle of the algorithm to identify sites and sequential order of metabolic regulations from sequential metabolomics data. a) Principle of the algorithm to identify sites and sequential order of regulations from metabolomics data with sequential structure. The metabolomics data gets split into individual frames, for which the regulated reactions get identified through metabolic network segmentation. (b) Structure of the Markov random field model for sequential data. For each frame of a sequential dataset a Markov random field model for univariate comparison is introduced (Fig 1b). To enforce a probabilistic dependency between hidden variables of neighboring sequence frames, the model includes additional sequence factor potential (*ψ*_*s*_) connecting adjacent hidden variables.

Metabolism provides the molecular building blocks, energy, and redox equivalents to fulfill the physiological needs of a cell. Therefore, a given metabolic perturbation must be compensated by other metabolic branches to sustain the cell’s physiological requirements. This means that a metabolic perturbation can cause primary regulation events, i.e., the enzymatic target of a perturbation, and secondary regulation events, i.e., the metabolic regulation events required to compensate for the effects of the perturbation. Thus, in metabolomics research, it is often crucial to know which enzymes are regulated, and in addition, the causal order of these regulation events. Differences in the sequence of regulation events given dynamic data, called sequential order from here, can give a first indication for causal regulatory interactions For example, the timing of regulations can distinguish between primary and secondary effects of a drug. However, to date, no method is available that can automatically infer the sequence of regulation events. To enable such analyses, we extended our approach for identifying sites to include identifications of the sequential order of metabolic regulation steps, based on dynamic metabolomics data, such as time courses or compound dilution series (Fig 2a). Specifically, the sequential data was split into individual frames, and each frame was represented by a MRF model for univariate data, as described previously (Figs 1b and 2b). We assumed that, without perturbation, the levels of a given metabolite at two consecutive data points were invariant. Therefore, we introduced a dependency on sequential data points with a sequence factor potential function ψ_S_(y_i,s-1_,y_i,s_), which connected hidden variables of neighboring frames (Fig 2b), as follows:

$$\psi_{S}\left(y_{i,s-1},y_{i,s}\right)=exp\left(-\lambda_{2}\cdot f\left(y_{i,s-1},y_{i,s}\right)\right)\;with\;f\left(y_{i,s-1},y_{i,s}\right)=\;\{\begin{array}{cc} -1 & if\;y_{i,s-1}=y_{i,s}\\ 1 & if\;y_{i,s-1}\neq y_{i,s} \end{array},$$

where λ_2_ is a weighting factor that quantifies the influences of neighboring sequence frames on each other. Given this sequence dependency, the conditional likelihood was defined as:

$$p\left(y|x\right)=\frac{1}{Z\left(x\right)}\cdot \overset{S}{\underset{s=1}{\prod }}\left(\overset{C}{\underset{j=1}{\prod }}exp\left(-\lambda_{1}\cdot \left(\frac{unique\left(c_{j,s}\right)-1}{size\left(c_{j,s}\right)}\right)\right)\cdot \overset{M}{\underset{i=1}{\prod }}exp\left(-\frac{{\left(x_{i,s}-\mu \left(y_{i,s}\right)\right)}^{2}}{2\sigma {\left(y_{i,s}\right)}^{2}}\right)\right)\cdot \overset{S}{\underset{s=2}{\prod }}\overset{M}{\underset{i=1}{\prod }}exp\left(-\lambda_{2}\cdot f\left(y_{i,s-1},y_{i,s}\right)\right),$$

where S is the total number of sequence frames, y_i,s_ and x_i,s_ are the hidden and observed variables, respectively, and c_j,s_ is the maximal neighborhood clique for each sequence frame, s. Similar to the univariate approach, we aimed to enable automatic identifications of sequences and neighborhood fractures with a scanning process (S3 Fig). First, the ranges of the neighborhood and the sequence weights, λ_1_ and λ_2_, were defined individually by exponentially increasing λ_1_ with constant λ_2_ = 0, until no more neighborhood fractures were identifiable, and by exponentially increasing λ_2_ with constant λ_1_ = 0 until no more fractures were found. Second, the neighborhood and sequence fractures were extracted by inference of the hidden state labels for every parameter combination, during a scan through all combinations of the defined ranges of λ_1_ and λ_2_.

Similar to the identification of the sites of metabolic regulation, we expected sequence fractures to be more relevant when they persisted with high sequential influence (i.e., high values of λ_2_). In the identification of significant fractures, we balanced the interdependency of the neighborhood, observation, and sequence factor potential functions, with a score function:

$$score\left(\lambda_{1},\lambda_{2},x|w_{t},w_{n}\right)=\overset{M}{\underset{i=1}{\sum }}\psi_{O}\left({\hat{y}}_{i}\left(\lambda_{1},\lambda_{2},x_{i}\right)\right)-w_{s}\frac{\#fractures_{sequence}\left(\hat{y}\left(\lambda_{1},\lambda_{2},x\right)\right)}{\text{max}\left(\#fractures_{sequence}\right)}-w_{t}\frac{\#fractures_{neighborhood}\left(\hat{y}\left(\lambda_{1},\lambda_{2},x\right)\right)}{\text{max}\left(\#fractures_{neighborhood}\right)},$$

where $\hat{y}\left(\lambda_{1},\lambda_{2},x\right)$ was the optimal module label distribution derived by inference, given λ_1_, λ_2_; and the observations **x**, $\#fractures_{sequence}\left(\hat{y}\left(\lambda_{1},\lambda_{2},x\right)\right)$, and $\#fractures_{neighborhood}\left(\hat{y}\left(\lambda_{1},\lambda_{2},x\right)\right)$, were the sequence and neighborhood fracture counts, respectively; given the inference solution, $\hat{y}$, the max(#fractures_*sequence*_) and max(#fractures_*neighborhood*_), represented the total counts of possible fractures, given the model structure; moreover, w_s_ and w_n_ were the weights that determined the influence of the numbers of sequence and neighborhood fractures in the score function. This score function served to balance the fracture frequency, to ensure that it was comparable between different experiments. By maximizing the score function, the best combination, ${\hat{\lambda }}_{1},{\hat{\lambda }}_{2}$, could be derived as follows:

$${\hat{\lambda }}_{1},{\hat{\lambda }}_{2}=\underset{\lambda_{1},\lambda_{2}}{\text{argmax}}\left(score\left(\lambda_{1},\lambda_{2},x|w_{s},w_{n}\right)\right).$$

Parameterizations were excluded from the maximization, when they gave a zero value for either the observation potential or the number of fractures. Thus, the sites and the sequential order (i.e., timing or sensitivity) of metabolic regulation steps that were represented by the most stable sequence and neighborhood fractures could be extracted by scanning through the increasing weights, w_s_ and w_n_.

## Results

### Parameter optimization for automatic identification of sites of metabolic regulation

**Fig 3:**
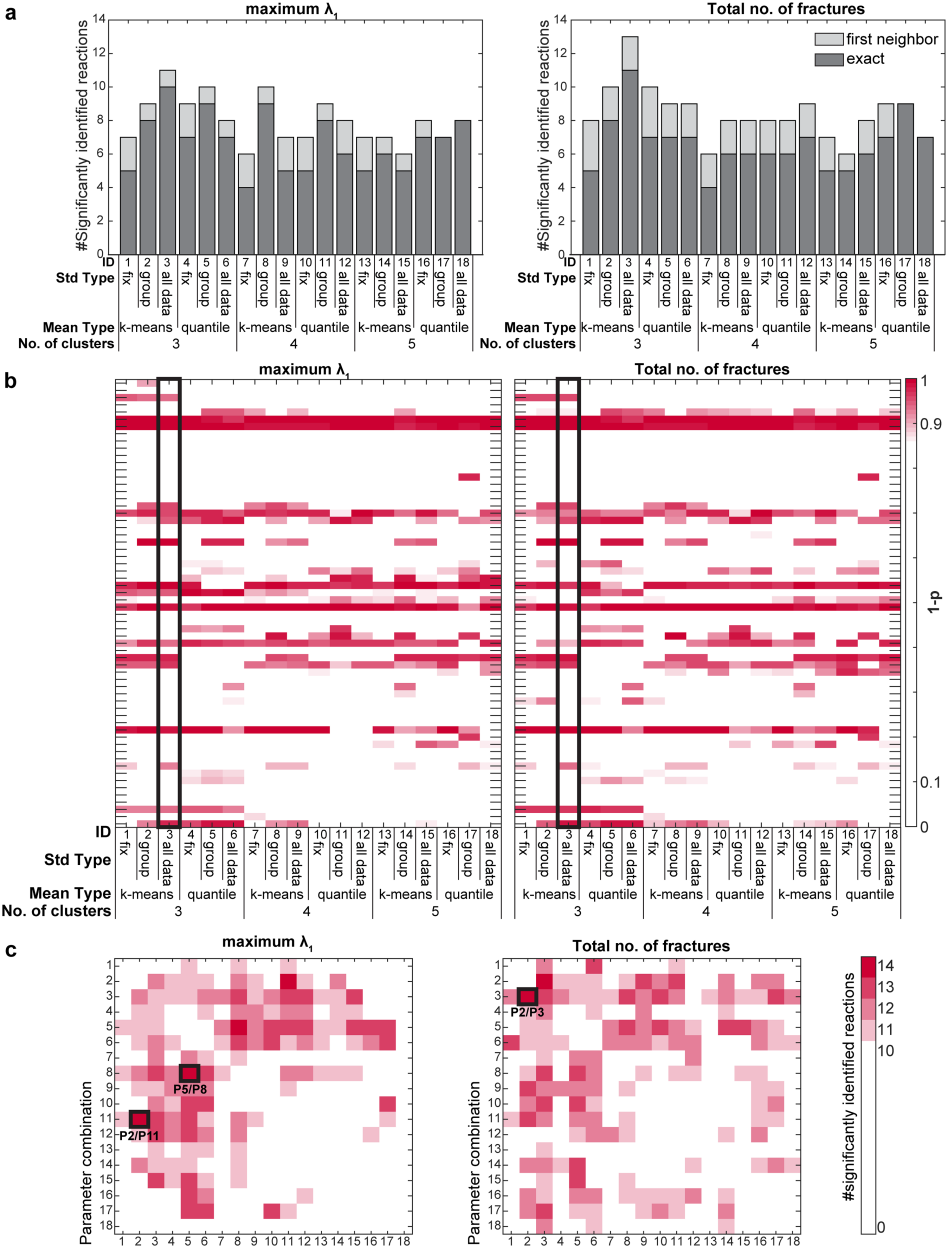
Parameter optimization for the accurate identification of experimentally perturbed reactions from metabolomics data. (a) Number of significantly identified reactions (p < 0.05) for each parameter combination for the reaction ranking based on maximum λ_1_ or total numbers of identified fractures (Details see S1 and S2 Text, S1 and S2 Figs). Significantly identified reactions were classified into “exact” if the experimentally perturbed reaction was inferred by the algorithm or as “first neighbor” if one of the first neighbor reactions of the perturbed reaction was inferred. P-values are calculated by a permutation test of the reaction labels with 1000 permutations. (b) Influence of the models parameter settings on the significance of the inference of individually perturbed enzymes. Black box marks the best single parameter combination (3 hidden states, mean type: k-means, std type: all data). Certain perturbed enzyme knock-outs (e.g. shdB, aldA) cannot be identified with the best parameter combination but with others (e.g. 3 hidden states, mean type: quantile, std type: all data). (c) Number of significantly identified reactions (p < 0.05) for rank product combinations of different parameter settings. Three combinations of two predictors with different model parameterizations (black boxes) improve the significantly inferred enzymes to 14. P-values are calculated by a permutation test of the reaction labels with 1000 permutations.

We first optimized the parameters used by the MNS algorithm (S1 Table), based on a non-targeted metabolomics dataset of 62 *Escherichia coli* single enzyme knockout and overexpression mutants, which exhibited a wide variety of metabolome phenotypes (S2 Table, details in S1 Text). The optimization results demonstrated that, for most parameter combinations, the MNS algorithm achieved inferences of regulatory sites that were comparable to those achieved by an expert scientist that manually identified the perturbed enzymes (S3 Table, S4 Fig). Furthermore, we evaluated whether the algorithm could provide a significantly better prediction of which enzyme was perturbed compared to a random guess with a permutation test. The results demonstrated that the MNS algorithm with the best parameterization (P3, S1 Table) identified 11 (maximum λ_1_) and 13 (#fractures) of the 62 perturbed enzymes, significantly better than the permutation test (Fig 3a, S3 Table).

Interestingly, when we performed a comparison of the performance of our approach with different individual parameter combinations, we found that, for some enzymatic perturbations (e.g., the *sdhC* knockout), a different parameter set was preferable (Fig 3b). Inspired by the notion that a combination of multiple predictors might improve the prediction results [29–32], we combined independent predictions obtained with different parameters, by integrating them with the rank product. When we tested the performance of these pairwise combinations, we found that three individual combinations of the parameterizations slightly improved the prediction results, and 14 (22.6%) reactions were identified significantly better with the MNS algorithm than with the permutation test (p <0.05, permutation test; Fig 3c, S3 Table).

### Performance evaluation and comparison to existing methods

We compared the performance of the MNS algorithm with optimized parameters against two state-of-the-art, but conceptually simpler, methods: reporter reactions [16] and mass-action ratios [15]. These comparisons employed independent metabolomics data from a genome-wide screen of 647 *E*. *coli* single enzyme knockout mutants [33]. We evaluated the prediction performance, based on the rankings of exact identifications of the perturbed reactions and of near misses, i.e., a prediction of the first-neighbor reaction. To evaluate significance, we performed a permutation test. The reporter reaction algorithm predicted 48 (7.4%) knocked-out enzymes significantly, and thereby surpassed the mass-action ratio algorithm, which identified 31 (4.8%) knocked-out enzymes significantly (p <0.05, permutation test; Table 1, S5 Fig). With the best single set of parameters derived from the previous optimization (P3), our MNS algorithm predicted 55 (8.5%) of the perturbed enzymes significantly; moreover, with the best combined parameterization (P2/P3/#fractures), our algorithm identified 74 (11.4%) of the knocked-out enzymes significantly (p <0.05, permutation test; Table 1, S5 Fig). Thus, our method showed a more than 50% improvement in prediction performance compared to current state-of-the-art-methods (Table 1, S5 Fig).

**Table 1:** Comparison of algorithms in the identification of the experimentally perturbed reactions in 647 *E*. *coli* enzyme knockout mutants. Significantly identified reactions were determined by a permutation test of the reaction labels with 1000 permutations and a p-value cutoff of 0.05.

Algorithm	Genes found in TOP10 Ranks [%]		#Significantly identified reactions		Significantly identified reactions [%]	
	Exact	Total	Exact	Total	Exact	Total
MNS—P3—max(λ1)	4.0	38.6	48	55	7.4	8.5
MNS—P3—#fractures	4.2	38.8	48	55	7.4	8.5
MNS—P2 & P11—max(λ1)	4.3	38.9	66	71	10.2	11.0
MNS—P5 & P8—max(λ1)	3.6	28.4	62	67	9.6	10.4
MNS—P2 & P3—#fractures	5.6	42.2	69	74	10.7	11.4
reporter reactions	2.8	43.6	37	48	5.7	7.4
mass action ratio	1.5	38.5	26	31	4.0	4.8

### Inference of regulatory sites in *E*. *coli* transcription factor knock-out mutants

To illustrate further the potential of the MNS approach, we applied it to three cases with widespread, complex metabolome changes, namely the *E*. *coli* transcription factor knockout mutants, Crp, MetR, and ArgR. For Crp, the reactant pairs predicted by the algorithm significantly overlapped with known targets of transcription factors (38% overlap, p = 0.023, hypergeometric test, S6 Fig) [34]. In contrast, for ArgR and MetR, no overlap was detected between the predicted and known targets (S6 Fig). The ArgR knockout should induce active arginine biosynthesis, even when arginine was present in the media [35]; however, we did not expect a significant overlap between known and predicted targets in MetR mutants, because methionine in the medium could inhibit MetR activity in wild-type *E*. *coli* [36].

**Fig 4:**
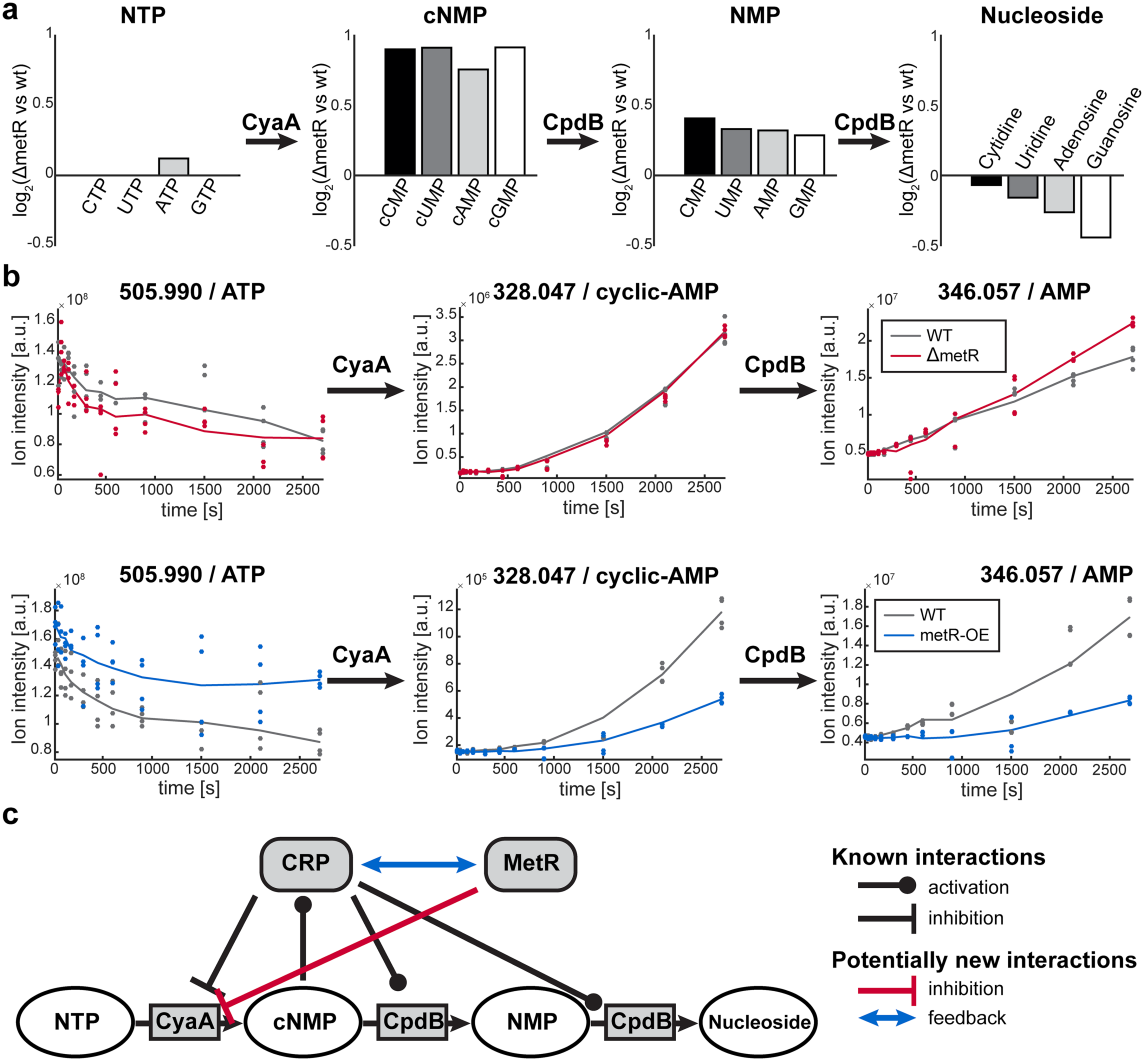
Identification of novel regulatory events in nucleotide metabolism mediated by MetR. (a) Change in nucleotide triphosphate (NTP), cyclic nucleotide monophosphates (cNMP), nucleotide monophosphates (NMP) and nucleoside metabolite levels comparing Δ*metR* knockout and wildtype *E*. *coli*. (b) Results of CyaA enzyme assays with 10 mM ATP as substrate in crude extracts of Δ*metR* knockout, *metR* overexpression and wildtype *E*. *coli*. (c) Known and potentially new interactions involved in the regulation of nucleotide metabolism. Our study suggests that MetR inhibits CyaA. This could be mediated through direct inhibition or indirect feedback for example to CRP, the known regulator of CyaA expression.

Surprisingly, results for the *metR* knockout mutant repeatedly identified *cyaA* and *cpdB* among the top predicted genes regulated (S4 Table). Both the CyaA and CpdB enzymes are involved in the homeostasis of cyclic nucleotide monophosphates (cNMPs), and they are known to be regulated by Crp, but not by MetR [37, 38]. Consistent with MNS predictions, the raw metabolite data revealed increased metabolite levels of all cNMPs and nucleotide monophosphates (NMPs), but nucleotide triphosphate (NTP) levels remained constant, and nucleoside levels declined (Fig 4a). This pattern suggested an activation of the adenylate cyclase, CyaA, or the inhibition of the 2',3'-cyclic-nucleotide 2'-phosphodiesterase/3'-nucleotidase, CpdB, by MetR. A non-specific effect on cNMP production rates could be excluded (S7a Fig). To verify the predicted regulation of CyaA and CpdB activity by MetR, we performed CyaA and CpdB enzyme assays in crude protein extracts of *E*. *coli metR* knockout and overexpression mutants. In extracts from *E*. *coli* with *metR* gene knockouts, we found elevated CyaA activity and unaltered CpdB activity (Fig 4b, S7b and S7c Fig). In *metR* overexpressing strains, we also found no significant change in enzymatic CpdB activity, but a strong reduction in enzymatic CyaA activity. These results confirmed that MetR directly or indirectly negatively regulated CyaA activity. In contrast, the predicted regulation of CpdB could not be confirmed. We speculated that either the prediction of CpdB could be considered a near miss in CyaA regulation or that *in vivo* regulation of CpdB activity could not be reproduced with *in vitro* enzyme assays. This latter possibility could occur, for example, when the regulation is mediated by allosteric interactions or post-translational modifications. Nevertheless, in summary, this example also demonstrated that novel sites of metabolic regulation could be identified by MNS, even in complex situations, like transcription factor knock-out mutants.

### Identification of the sequential order of oxidative stress-induced metabolic regulation in human fibroblasts treated with increasing concentrations of H_2_O_2_

As mentioned before, it is often crucial that, in addition to identifying sites, a model can also determine the sequential order of metabolic regulatory steps. For example, it is often important to distinguish between the primary targets and the secondary effects of a metabolic drug. Therefore, we investigated the potential of our MNS algorithm in predicting both the sites and sequential order of metabolic regulation, based on dynamic metabolite data. We applied the algorithm to a previously published metabolomics dataset from fibroblasts treated with increasing concentrations of H_2_O_2_ [39]. We ran the algorithm with three clusters that had fixed mean values (μ_1_ = -0.1, μ_2_ = 0, μ_3_ = 0.1), with data-dependent standard deviation values for the observation function, and with a fixed scanning range, for λ_1_ and λ_2_, which was determined with a prior, automatic coarse-grained scan through the parameters (S2 Text). The score distributions, which depended on sequence and neighborhood weights, w_s_ and w_n_, indicated that the influence of the metabolic neighborhood, λ_1_, and of the sequential hidden variables, λ_2_, could be balanced to filter out equal degrees of non-significant neighborhood and sequence fractures (S8 Fig). The application of the algorithm smoothed the heterogeneous module label distributions with increasing neighborhood and sequential influences (λ_1_ and λ_2_), which resulted in a continuous reduction of sequence and neighborhood fractures, and a focus on a small subset of metabolites and reactions (S8, S9 and S10 Figs). For certain ranges of the neighborhood and sequential weights, the analysis resulted in a pseudo steady-state distribution of the module labels and fractures (grey area, S8b Fig). We expected that these pseudo steady-states would be of special interest, because they represented module label configurations with a certain stability, and thus, they might be biologically meaningful.

**Fig 5:**
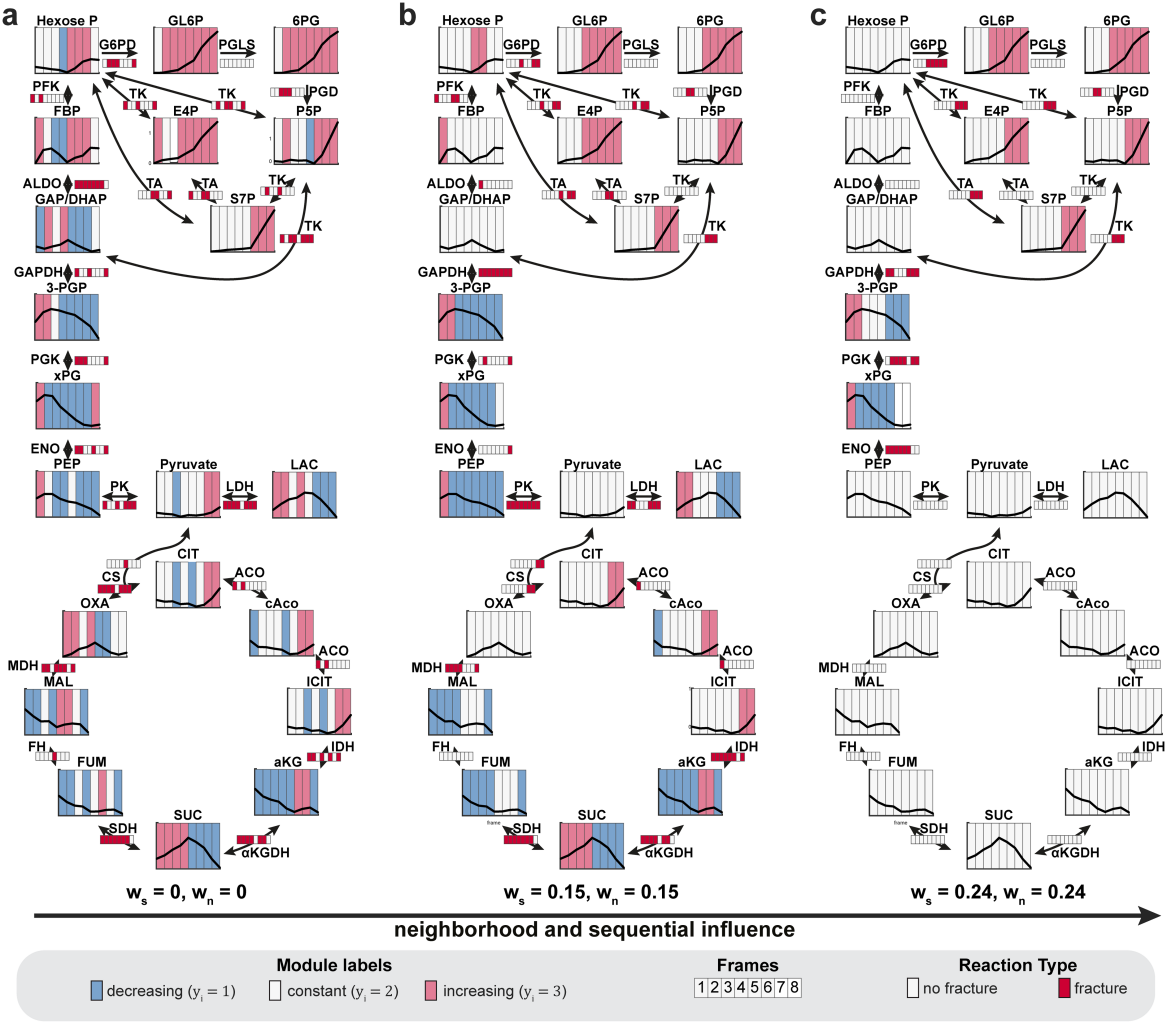
Identification of sequential order of oxidative stress induced metabolic regulations in fibroblasts. Application of MNS algorithm for sequential data on metabolomics data from fibroblasts treated with increasing concentrations of H_2_O_2_. The figure shows segmentation results with increasing incluence of the neighborhood and sequential dependency: (a) sequence weight w_s_ = 0, neighborhood weight w_n_ = 0, (b) w_s_ = 0.15, w_n_ = 0.15, (c) w_s_ = 0.24, w_n_ = 0.24. In each time profile inset, the black line reports the log2 of the fold-change relative to the first time point (frame). With increasing dependency to the neighborhood and sequence, only the major known regulators involved in the metabolic reponse to oxidative stress remain as inferred regulatory sites. Furthermore, the algorithm correctly infers the sequential order of an initial activation of G6PD and inhibition of glycolytic flux (GAPDH) which is followed by a rerouting of flux into P5P, S7P, and E4P via PGD and back to upper glycolysis via TK and TA [39]. Abbreviations: Hexose P: hexose phosphates, GL6P: gluconolactone 6-phosphate, 6PG: 6-phospho gluconic acid, FBP: fructose bisphosphate, E4P: erythrose 4-phosphate, S7P: sedoheptulose 7-phosphate, P5P: pentose 5-phosphates, GAP/DHAP: glyceraldehyde 3-phosphate/dihydroxyacetone phosphate, 3-PGP: 3-phosphoglyceroyl phosphate, xPG: 2/3-Phosphoglyceric acid, PEP: phosphoenolpyruvate, LAC: lactic acid, CIT: citric acid, cAco: cis-aconitic acid, ICIT: isocitric acid, aKG: α-ketoglutaric acid, SUC: succinic acid, FUM: fumaric acid, MAL: Malic acid, OXA: oxaloacetic acid, G6PD: glucose-6-phosphate dehydrogenase, PGLS: 6-phosphogluconolactonase, PGD: phosphogluconate dehydrogenase, TK: transketolase, TA: transaldolase, PFK: phosphofructokinase, ALDO: aldolase, GAPDH: glyceraldehyde 3-phosphate dehydrogenase, PGK: phosphoglycerate kinase, ENO: enolase, PK: pyruvate kinase, LDH lactate dehydrogenase, CS: citrate synthetase, ACO: aconitase, IDH: isocitrate dehydrogenase, αKGDH: α- ketoglutarate dehydrogenase, SDH: succinate dehydrogenase, FH: fumarate hydratase, MDH: malate dehydrogenase.

Next, we further investigated the biological relevance of the inferred metabolic regulatory steps. At an intermediate level of neighborhood and sequential influence, we identified oxidative stress-induced regulatory steps in the citric acid cycle, glycolysis, and pentose phosphate pathway (Fig 5b). Most predicted enzymes in the citric acid cycle, including succinate, α-ketoglutarate, and malate dehydrogenase, were previously reported to be sensitive to oxidative stress [40–42]. Isocitrate dehydrogenase was not previously reported to be influenced by oxidative stress, but the upstream enzyme, aconitase, was known to be inhibited by oxidation [40]; however, our algorithm did not predict that aconitase was regulated. We hypothesized that this discrepancy was due to the inability of the non-targeted metabolomics method to distinguish between isomers like citrate and isocitrate, which are the substrate and product, respectively, in the aconitase-catalyzed reaction [43]. To test this hypothesis, further follow up analysis must be performed with targeted mass spectrometry methods that enable a distinction between the two isomers.

At high sequential and neighborhood influences, the only inferred metabolic regulations were the major, known regulators of the metabolic response to oxidative stress in upper glycolysis and the pentose phosphate pathway (Fig 5c, S9 and S10 Figs). These inferred regulations included oxidative stress-mediated activation of glucose 6-phosphate dehydrogenase [39], inhibition of glycolytic flux by oxidation of glyceraldehyde-3-phosphate dehydrogenase [39, 44, 45], and changes in the directions of net fluxes for transketolase and transaldolase [39]. Importantly, our algorithm revealed that the activation of glucose 6-phosphate dehydrogenase and the inhibition of glycolytic flux occurred at equally low H_2_O_2_ concentrations, and that the accumulation of non-oxidative pentose phosphate pathway metabolites was observed only at high H_2_O_2_ concentrations (Fig 5c). Because the accumulation of pentose phosphates is a consequence of increased glucose 6-phosphate dehydrogenase flux [39], our method correctly identified the sequential order of these regulatory events.

## Discussion

**Fig 6:**
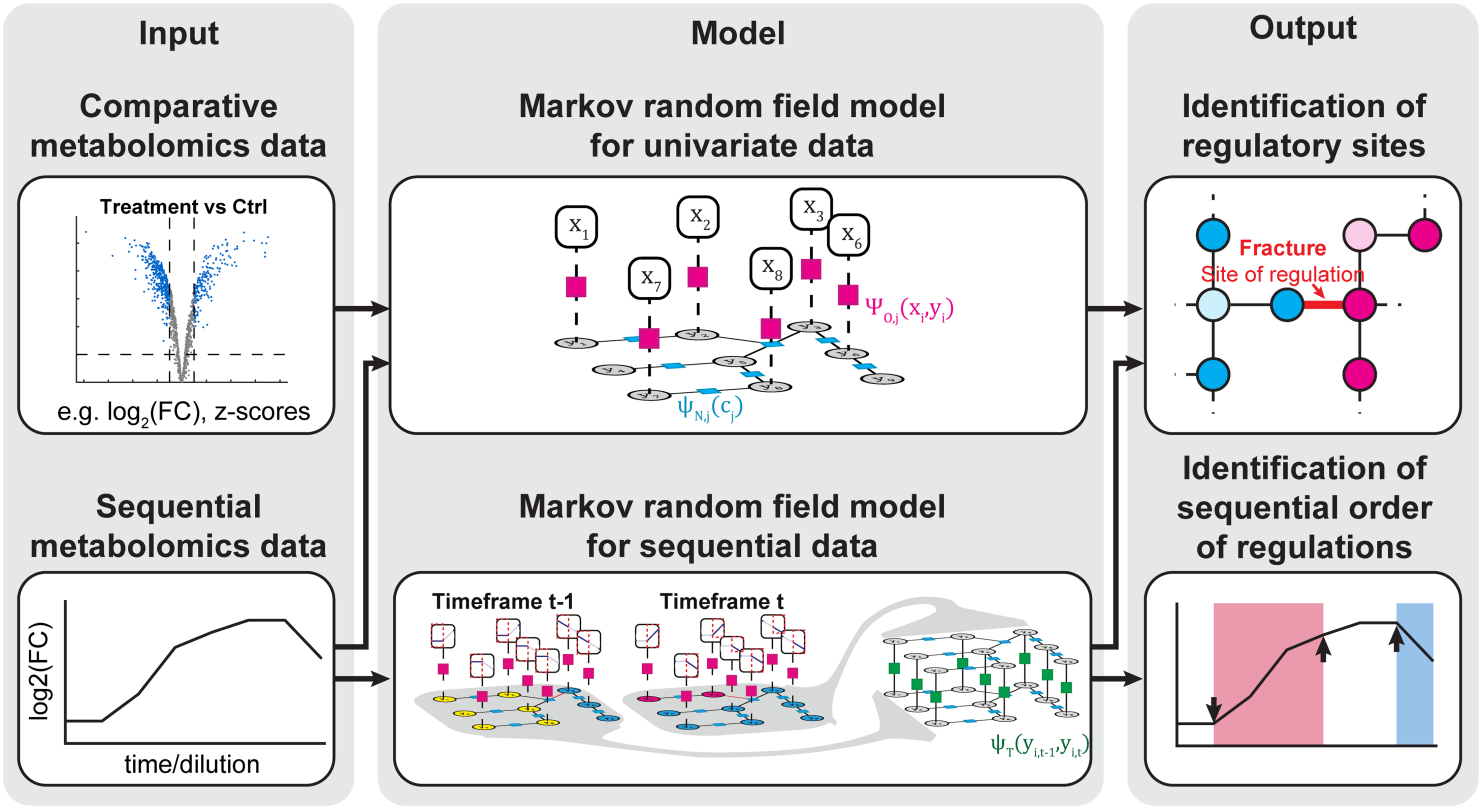
Overview of the MNS toolbox. The MNS algorithm enables to predict sites and the sequential order of metabolic regulations from metabolomics data.

Here, we presented the MNS algorithm, a novel computational method that employs probabilistic graphical models to infer the sites and sequential order of metabolic regulatory events, based on relative metabolomics data and metabolic network topology (Fig 6). Exploiting probabilistic dependencies specified by the metabolic model, our approach successfully coped with large-scale, noisy, and sparse datasets. In addition, our approach outperformed existing methods, like reporter reactions [16] and mass-action ratios [15], in the identification of regulatory sites. Furthermore, it could infer the sequential order of regulatory events, which is often essential for identifying direct and indirect metabolic regulatory events.

### Constraints on the application of the method

In the parameter optimization and method comparison sections, we demonstrated that, although the MNS algorithm inferred many genetically perturbed enzymes correctly, a significant portion of perturbed enzymes were not identified. However, the algorithm’s prediction performance was comparable to a manual analysis by an expert. A low prediction performance might be due the fact that some genetic perturbations of enzymes have little effect on metabolism. For example, inhibition of an enzyme that carries no metabolic flux will not influence the metabolic phenotype. Likewise, when a flux-carrying enzyme is perturbed, concomitant regulation of the involved isoenzyme can maintain the flux of the perturbed reaction. In both cases, the metabolite data will not indicate any changes, and the perturbed reaction will not be identifiable.

Moreover, the perturbation of a single metabolic enzyme can cause global rearrangements in metabolism. This is due to the fact that metabolism supplies the cell with the molecular building blocks, energy, and redox equivalents it needs to meet the cell’s physiological requirements. Therefore, perturbations of required enzymes can induce secondary regulation to stimulate other metabolic branches that can compensate for the changed flux. Given steady state data, these secondary regulation events might not be distinguishable from the regulation of the experimentally perturbed enzyme, or in some cases, secondary events might occlude the perturbation. Therefore, it is highly important to identify the causal chain of regulatory events that occur after a metabolic perturbation. The extended version of the metabolic network segmentation algorithm, for sequential metabolomics data enables to identify differences in the sequence of regulations, e.g. a temporal delay in time course experiments. These differences in the sequential order of regulations can be causal, but also result from secondary effects. For example, a drug can perturb the activity of several enzymes in parallel, but due to differences in turnover rates of the enzymes the regulations might be observed with time delays. Therefore, the sequential order would indicate that there are secondary effects although the enzymes are regulated directly by the drug. Therefore additional analysis are required to increase certainty about causality in the chain of regulations. For example, dynamic simulations of detailed mechanistic models of biological subnetworks can indicate if the predicted dependencies amongst regulations can explain the observed data. Furthermore, additional experiments with enzyme knockouts enable to investigate the dependency of potential secondary regulations on primary ones in enzyme knockout experiments.

Moreover, it is necessary to ensure that the sequential samples, for example dilution steps in a drug dilution series or the sampling frequency in time course experiments, have sufficient resolution to distinguish the regulatory events. Notably, modern sampling approaches reach a sampling frequency in the range of seconds. Although some metabolic regulations can occur even with delay times below seconds, these approaches meet the requirements for distinguishing the sequence of most metabolic regulatory events [11, 46].

### Plans for further development of the algorithm

Due to the modularity of the underlying MRF models, the presented MNS algorithm can be extended and used for other applications. One problem in metabolomics research is the annotation of metabolites in non-targeted datasets. Recently, different network-based approaches have demonstrated that information on the metabolic environment improved the annotation of metabolites [18, 47, 48]. We think that our method might also be extended to include uncertainty in metabolite annotation. This extension would require that we add to the MRF models a third node-type, which represents the raw data features. The raw-data nodes would be connected to potential metabolites with a given probability, which would describe how likely the connection was between the raw-data feature and the metabolite. During the segmentation process, this probability could be continuously optimized to identify sites of regulation steps and to identify likely connections between metabolites and raw-data features.

Furthermore, we are currently working on methods for focusing on the extraction of co-regulated enzyme-metabolite modules from integrated ‘omics’ data. Recently, different subgraph extraction approaches have achieved great success in identifying areas in the metabolic network that showed changing activity [49–51]. These subgraphs have simplified the complex data structure, which has enabled manual interpretations within a network context. We aim to design an algorithm that can identify co-regulated metabolite-enzyme modules in metabolomics data integrated into metabolic models, together with multiple other ‘omics’ layers that might influence enzymatic activity; for example, data from transcriptomics, proteomics, or post-translational modification analyses. This new algorithm would provide a means to make direct inferences from the extracted modules to determine which type of regulation was most likely to cause changes in metabolic activity.

One further promising extension we are planning to implement is a multidimensional clustering approach for multivariate datasets. This approach would aim to form clusters of metabolite levels, datasets, and the metabolic network, in parallel. In contrast to current approaches (e.g., correlation networks [17, 52]), this algorithm would enable the identification metabolic modules of metabolites and their corresponding datasets, simultaneously. Due to the high metabolic heterogeneity in cancers [53–55], such a method would be highly advantageous for identifying similarly regulated metabolic branches in different cancer cell lines or tumor samples. This could facilitate tumor categorizations and improve the development of tumor-specific treatments that target the metabolism.

### Conclusion

In its current state, the MNS method is broadly applicable to different metabolic setups, where it can facilitate automated interpretations of large-scale metabolomics data. Moreover, due to the modularity of the underlying MRF models, more advanced metabolomics data analysis approaches can be developed, based on the MNS algorithm. Thus, the presented MNS approach will enhance our understanding of metabolism and its interactions with cellular signaling and regulatory processes.

## Materials and methods

### Manual identification of perturbed reactions in *E*. *coli* enzyme mutants using visual integration

To get a gold standard how well the perturbed reactions given the metabolomics data of *E*. *coli* enzyme knockout and overexpression mutants can be identified by experts, we extract for each mutant a local network comprising metabolites and reactions with a maximal distance of three reactions around the perturbed reaction and visualized the log_2_(fold-changes) comparing metabolome data from knockout and wildtype strains on the individual metabolites using Cytoscape v3.0 [56]. Based on this subnetwork, two individual experts guessed by eye which of the reactions was perturbed without knowing the enzyme and metabolite names. The datasets were then classified into three classes based on the distance between the manual guess and the genetically perturbed enzyme. The classes are exact (distance = 0), first neighbor (distance = 1) and not identifiable (distance > 1).

### Parameter settings of the univariate MRF model for the parameter optimization for the identification of sites of metabolic regulation

In the parameter optimization for the automatic inference of sites of metabolic regulations, we optimized three parameters, the number of hidden state labels, the mean values and the standard deviations of the observation potential functions. The hidden state labels were varied between 3, 4 and 5. The hidden state label dependent mean values were either set to the mean values of the clusters derived k-means clustering with Euclidean distance (k-means) or set to values equally distributed between the 0.001 and 0.999 percentile of the input data to guarantee homogenously distributed centers of the observation potential functions (quantile). The hidden state label dependent standard deviations was set identical for each hidden state label either to 1 (fix), to the average standard deviation of the k-means clusters (k-means), or to the standard deviation of the complete data set. This resulted in total in 18 parameter combinations (S1 Table).

To identify the best Markov random field model parameterization, we inferred for each parameter combination 62 known regulatory sites using metabolomics data from 62 *E*. *coli* enzyme knock-out and overexpression mutants. We determined significantly identified reactions by comparing the real inferred rank of the perturbed reaction and its first neighbors with the rank distribution of the perturbed reaction and its first neighbors given 1000 permutations of the reaction labels resulting in p-values for each perturbed reaction and each method. Thereby we identified how many known perturbed reactions were identified correct.

### Method comparison for the inference of sites of metabolic regulation

We compared the potential of our algorithm to identify sites of metabolic regulation with reporter reactions [16] and mass-action ratio [15]. We implemented the reporter reaction and the mass-action ratio as described previously, considered only the direct substrates and products of a reaction and replaced undetected metabolites by random sampling of not annotated peaks [15, 16]. For both methods all reactions in the model were ranked according to descending absolute z-scores (reporter reactions) or descending absolute mass-action ratio. For all methods including ours we determined significantly identified reactions by comparing the real rank of the perturbed reaction and its first neighbor reactions with the rank distribution of the perturbed reaction and its first neighbors given permutations of the reaction labels resulting in p-values for each perturbed reaction and each method. The p-values for reactions identified exactly or to the first neighbor were calculated individually. A reaction was considered significantly identified for p-values < 0.05 of the exact and/or first neighbor identification.

### Strains and overnight pre-culture preparation

All used *E*. *coli* knock-out strains were part of the KEIO knock-out library [57] and all used overexpression strains were part of the ASKA overexpression library [58]. KEIO strains were compared against *E*. *coli* K12 BW25113, denoted as KEIO WT. Induced ASKA strains were compared against non-induced ASKA strains, denoted as ASKA WT or against the average over all induced ASKA strains per plate, denoted as others. For overnight precultures, strains were inoculated from LB rich medium agar plates (10 g/L Bacto peptone, 5 g/L Bacto yeast extract, 5 g/L NaCl, with additional 15 g/L agar-agar for solidification) into 5 mL of LB liquid medium in 15 mL culture tubes and incubated for 16 h at 37°C and 300 rpm. LB medium for KEIO strains was supplemented with 50 μg/mL kanamycin as resistance marker. KEIO WT was cultured in pure LB medium. ASKA strains were supplemented with 20 μg/mL chloramphenicol as resistance marker. Overexpression in ASKA strains was induced by adding of 100 μg/mL IPTG. Overnight preculture preparation was conducted identical for all experiments.

### Growth phenotyping of enzyme mutant strains

To define optical density at 600 nm (OD) values corresponding to mid-log phase for KEIO and ASKA strains, growth experiment were conducted. KEIO and ASKA strains were inoculated at OD 0.05 from overnight precultures and subsequently cultured in 1 mL M9 medium (7.52 g Na_2_HPO_4_·2H_2_O, 3 g KH_2_PO_4_, 0.5 g NaCl, 2.5 g (NH_4_)_2_SO_4_, 14.7 mg CaCl_2_·2H_2_O, 246.5 mg MgSO_4_·7H_2_O, 16.2 mg Fe(III)Cl_3_·6H_2_O, 180 μg ZnSO_4_·7H_2_O, 120 μg CuCl_2_·2H_2_O, 120 μg MnSO_4_·H_2_O, 180 μg CoCl_2_·6H_2_O, 1 mg thiamine hydrochloride per liter of deionized water) supplemented with 4 g/L glucose and 2 g/L N-Z casein hydrolysate in 96-deep-well plates at 37°C and 300 rpm. To induce overexpression in ASKA strains, the medium was supplemented with 100 μg/mL IPTG, all other strains were cultured in unsupplemented medium. For each mutant, four technical replicates were used. OD was measured in intervals of 45 min for a total of 10 time points using a TECAN sunrise spectrophotometer. Based on the observed growth rates, strains for metabolomics experiments were selected (S2 Table).

### Extraction of the *E*. *coli* enzyme knockout and overexpression metabolome

Strains for metabolomics measurements were prepared identical as described for growth experiment of enzyme mutant strains. In total, 42 KEIO enzyme knockout strains, plus one KEIO WT per plate and 20 ASKA strains plus three non-induced ASKA WT strains per plate were inoculated. Each strain was grown to mid-exponential phase, i.e. OD 1.60 for KEIO strains and OD of 0.80 for ASKA strains. Harvested cells were centrifuged at 4000 rpm for 10 min at 0°C. Cell pellets were immediately extracted with 150 μL preheated ddH_2_0 for 10 min at 80°C. The extraction broth was centrifuged for 10 min with 4000 rpm at 0°C. The supernatants were diluted 1:5 in ddH_2_0 for non-targeted metabolomics.

### Generation of crude protein extracts for enzyme assays

For the generation of crude protein extracts ASKA MetR induced and non-induced strains were inoculated in 1:25 dilutions and KEIO WT and *metR* KO strains in 1:50 dilutions in M9 medium with N-Z casein plus 2 g/L, for induced strains additionally supplemented with 100 μg/mL IPTG, and grown to a final OD of 0.6 (ASKA) or 1.2 (KEIO). The cell suspensions were transferred into 50 mL falcon tubes and centrifuged at 4000 rpm for 10 min at 0°C. For protein extraction, supernatants were removed and pellets resuspended in 3.5 mL ice-cold Tris-HCI buffer (pH 7.5, 5 mM MgCl_2_, 2 mM DTT and 4 mM PMSF). Cells were subsequently lysed by a French press to obtain crude cell lysates. Enzyme concentrations were normalized by a colorimetric Bradford assay [59]. Total protein concentrations of extracts from KEIO strains were normalized to a protein concentration of 2.69 mg/mL and from ASKA strains to a total protein concentration of 1.47 mg/mL.

### CyaA and CpdB enzyme assays

To determine the enzyme activity of CyaA and CpdB in dependence of MetR activity, enzyme assays for the protein crude extracts were conducted with a final concentration of 1 mM or 10 mM of AMP, cAMP or ATP as substrates. Enzyme assays were conducted at 37°C with two biological replicates. For the enzyme assays 100 μL of pre-warmed crude cell extract were incubated with 50 μL of substrate. To study the enzymatic reaction dynamics 10 μL samples were taken at various time points (15 s, 45 s, 75 s, 120 s, 180 s, 300 s, 450 s, 600 s, 900 s, 1500 s, 2100 s, 2700 s) and immediately quenched in ice cold 100% methanol. To remove precipitated protein samples were centrifuged at 4000 rpm at 0°C for 10 min, and reactant concentrations in supernatants were measured by non-targeted metabolomics using a previously published method [43].

### Non-targeted metabolomics by flow injection—Time of flight mass spectrometry

Non-targeted analysis of metabolite extracts was performed by flow injection—time of flight mass spectrometry on a Agilent 6550 ion funnel QTOF instrument (Agilent, Santa Clara, CA) in negative mode 4 GHz, high resolution in a m/z range of 50–1000 as described previously [43]. A 60:40 mixture of isopropanol:water supplemented with NH_4_F at pH 9.0, as well as 10 nM hexakis(1H, 1H, 3H-tetrafluoropropoxy)phosphazine and 80 nM taurocholic acid for online mass calibration. Ions were annotated to metabolites based on exact mass considering [M-H]^-^ and [M+F]^-^ and 0.001 Da mass accuracy using the KEGG eco database [24]. All metabolomics data analysis was performed using Matlab 2014b (The Mathworks, Natick, MA).

## Supporting information

S1 Fig Workflow of the metabolic network segmentation (MNS) algorithm for univariate data to automatically identify sites of metabolic regulation. The algorithm integrates large-scale metabolomics data and metabolic models employing Markov random fields. The Markov random field model consist of a network of discrete hidden variables and of continuous observed variables for each detected metabolite. The (module) labels or states of the hidden variables describe to which module, i.e. a group of neighboring metabolites with similar metabolite changes, a certain metabolite belongs to. Observation potentials enforce a dependency between observations and the label of the hidden variables and neighborhood potentials enforce homogeneity of the module labels of neighboring metabolites, which is weighted by λ_1_. Given the metabolic observations and a certain model parameterization, the hidden state label distribution is inferred using the LazyFlipper algorithm from the OpenGM toolbox [25, 26]. The hidden state-label distribution is used to identify modules of similarly changing metabolites and the fractures between them, which we assume to represent sites of metabolic regulation. We infer the most relevant regulatory sites in a sequential scanning process with increasing influence of the neighborhood λ_1_. Since we assume that the most stable reactions represent biological meaningful regulations, we rank the reactions according to maximum λ_1_ at which a reaction is classified as fracture and according to the frequency of how often a reaction is classified as fracture. In case of combinations of multiple predictors with different parameterizations, the ranking is determined through rank products. Significance for a single predictor is assessed using a permutation test and for combination of predictors using significance test’s for rank products [28].
(TIF)

S2 Fig Example application of the metabolic network segmentation algorithm for univariate comparison to identify sites of metabolic regulation in H_2_O_2_ treated fibroblasts with transketolase knockdown (TKT KD) compared to wildtype. (a) Raw Data of the univariate comparison comparing metabolomics data from TKT KD treated with H_2_O_2_ to wildtype treated with H_2_O_2_. Data from [39]. (b) MNS inference results of module labels with increasing neighborhood influence λ_1_. With increasing λ_1_ only the perturbed reaction (TKT) and G6PD remain as inferred regulatory sites. (c,d) Overview of the (c) metabolite module labels and the (d) fractures. Arrows in (d) indicate how individual reactions are ranked #fractures and max λ_1_ to automatically infer regulatory sites (S1 Fig). Abbreviations: G6P: glucose 6-phosphates, F6P: fructose 6-phosphate, GL6P: gluconolactone 6-phosphate, 6PG: 6-phospho gluconic acid, FBP: fructose bisphosphate, E4P: erythrose 4-phosphate, S7P: sedoheptulose 7-phosphate, R5P: ribose 5-phosphate, Ru5-P: ribulose 5-phosphate, X5P: xylulose 5-phosphate, GAP/DHAP: glyceraldehyde 3-phosphate/dihydroxyacetone phosphate, BPG: Bisphosphoglyceric acid, 2/3PG: 2/3-Phosphoglyceric acid, PEP: phosphoenolpyruvate, PYR: Pyruvate, LAC: lactic acid, A-CoA: acetyl-CoA, GPI: glucose phosphate isomerase, G6PD: glucose-6-phosphate dehydrogenase, PGLS: 6-phosphogluconolactonase, PGD: phosphogluconate dehydrogenase, TKT: transketolase, TALDO: transaldolase, PFK: phosphofructokinase, ALDO: aldolase, TPI1: triosephosphate isomerase 1, GAPDH: glyceraldehyde 3-phosphate dehydrogenase, PGK: phosphoglycerate kinase, ENO: enolase, PK: pyruvate kinase, LDH lactate dehydrogenase, PGAM: phosphoglycerate mutase, RPIA: ribose 5-phosphate isomerase, RPE: ribulose 5-phosphate epimerase.
(TIF)

S3 Fig Workflow of the metabolic network segmentation algorithm to identify sites and sequential order of metabolic regulations from sequential data. The algorithm splits sequential metabolomics data into individual frames and introduces for each frame a Markov random field model for univariate data (Fig 1b). The hidden states between neighboring sequential frames are connected by a sequence factor potential, which is weighted by λ_1_, to enforce a dependency between sequential data points. Similar to the MNS model for univariate data, we infer the most relevant regulatory sites and their sequential order in a step-wise scanning process with increasing influence of the neighborhood λ_1_ and sequential frames λ_2_ (S1 Fig).
(TIF)

S4 Fig (a) Principle and (b) results of the visual inspection of the metabolomics data of 62 *E*. *coli* enzyme knockout and overexpression strains. (a) Given the metabolite data mapped on a subnetwork around the perturbed reaction (maximal three reaction steps), two individual users inferred the perturbed reaction by visual inspection. Reactions are categorized into “exact” if the users identified the experimentally perturbed reaction (e.g. tktA KO), “first neighbor” if the users inferred one of the first neighbor reactions of the perturbed reaction (e.g. fumC KO) or “> first neighbor” if the inferred reaction was more than one reaction step away from the perturbed enzyme or was not identifiable at all (e.g. carA OE). (b) Only about 5% of the perturbed reactions were identified exactly, about 60% one of the first neighbor reactions was inferred, and more than 30% of the reactions were not identifiable.
(TIF)

S5 Fig Comparison of multiple algorithms for (a) the number of significantly identified reactions and (b) the number of exactly identified reactions versus their rank. Independent of the parameterization the MNS algorithm outperforms current state-of-the-art methods in the identification of perturbed enzymes.
(TIF)

S6 Fig Identification of regulatory sites in 3 *E*. *coli* transcription factor knockout mutants using MNS algorithm. (a) Number of significantly identified reaction pairs (p < 0.01). Analysis was performed using a combination of the predictors with parameterization 2 and 3 (S1 Table). (b) Overlap between identified reaction pairs and reaction pairs that are known transcription factor targets. Ratio is determined comparing overlap and number of significantly predicted reaction pairs. p-value for Crp is calculated using a hypergeometric test.
(TIF)

S7 Fig (a) Growth and (b,c) enzyme assays of *metR* knockout and overexpression (MetR-OE) mutants. (a) Each bar represents mean values and standard deviation of doubling times at exponential growth phase of 4 individual biological replicates. There is no difference in doubling time comparing wild type E. coli and *ΔmetR* knockout mutants. (b,c) Enzyme assays with (b) 10 mM cAMP and (c) 10 mM AMP as substrate. Data shown represents mean values of 2 biological (except overexpression assays in c) and 2 technical replicates.
(TIF)

S8 Fig Detailed analysis of influence of neighborhood and sequential weights on fractures, observation potential and scores of the MNS model for sequential data. (a) Neighborhood and sequential influence λ_1_ and λ_2_ dependent distribution of sum neighborhood fractures, frame fracture and observation potential. (b) Total number of neighborhood and frame fracture with increasing penalization of fractures w_s_ and w_n_. Shaded areas indicate pseudo-steady states of the fracture frequency, i.e. the number of fractures is not increasing for a certain range of weights. These are can be considered as module label distributions with a certain stability. Dashed lines indicate the weight values used for further analysis. (c) Influence of increasing penalization of sequence and neighborhood fractures on score (score = Sum of observation factor potential—w_s_*sum of sequence fractures—w_n_*sum of neighborhood fractures). White stars in (c) indicate the maximal score for the setting of w_s_ an w_n_. With increasing penalization of fractures the optimal balance between number of fractures and the summed observation factor potential, i.e. how well do the model and the hidden module labels describe the data, can be determined. Score is normalized to a range between 0 and 1.
(TIF)

S9 Fig (a) Module labels and (b) sequence fractures of metabolites for increasing weights w_s_ and w_n_. With increasing influence from metabolic neighborhood w_n_ and sequential frames w_s_ the (a) module label distribution gets homogenized and only the most important (b) sequence fractures remain. Weight values were determined in S8b Fig.
(TIF)

S10 Fig Neighborhood fractures of reactions at different frames for increasing weights w_s_ and w_n_. With increasing influence from metabolic neighborhood w_n_ and sequential frames w_s_ the neighborhood fracture distribution gets homogenized and only the most important fractures remain. Weight values were determined in S8b Fig.
(TIF)

S1 Table Model parameterizations for the optimization of the MNS method. Description: No. of hidden states represents the number of different modules. Mean Type describes how the mean values of the observation potential function are derived (k-means: μ derived from the k-means clustering results; quantile: μ equally distributed between the 0.001 and 0.999 quantile of the data). Standard deviation type describes how the individual standard deviations of of the observation potential functions are determined (fix: fix standard deviation value for all hidden states **σ** = 1, k-means: individual σ for each hidden state equal to the standard deviation of the data points in a k-means cluster, all data: σ of all hidden states equal to standard deviation of the complete data).
(PDF)

S2 Table Overview of *E*. *coli* enzyme overexpression and knockout mutants used to test the performance of the MNS approach for univariate data to identify known sites of metabolic regulation. (PDF)

S3 Table Results of the identification of known regulatory sites from *E*. *coli* enzyme knockout and overexpression. Significantly identified reactions were determined by a permutation test of the reaction labels with 1000 permutations and a p-value cutoff of 0.05.
(PDF)

S4 Table Top predictions of MetR regulated reactions by the MNS algorithm. The analysis was performed with a combination of two parameterizations (S1 Table, P2 & P3).
(PDF)

S1 Text Details on the parameter optimization. (PDF)

S2 Text Details on the determination of λ_1_ and λ_2_ scanning ranges for the application of the MNS model for sequential data on hydrogen peroxide treated fibroblasts. (PDF)
