# Supplementary figures and images for: Metabolic network segmentation: A probabilistic graphical modeling approach to identify the sites and sequential order of metabolic regulation from non-targeted metabolomics data
Source: PLoS Comput Biol. 2017 Jun 9;13(6):e1005577. doi: 10.1371/journal.pcbi.1005577 (PMC5482507; doi:10.1371/journal.pcbi.1005577)

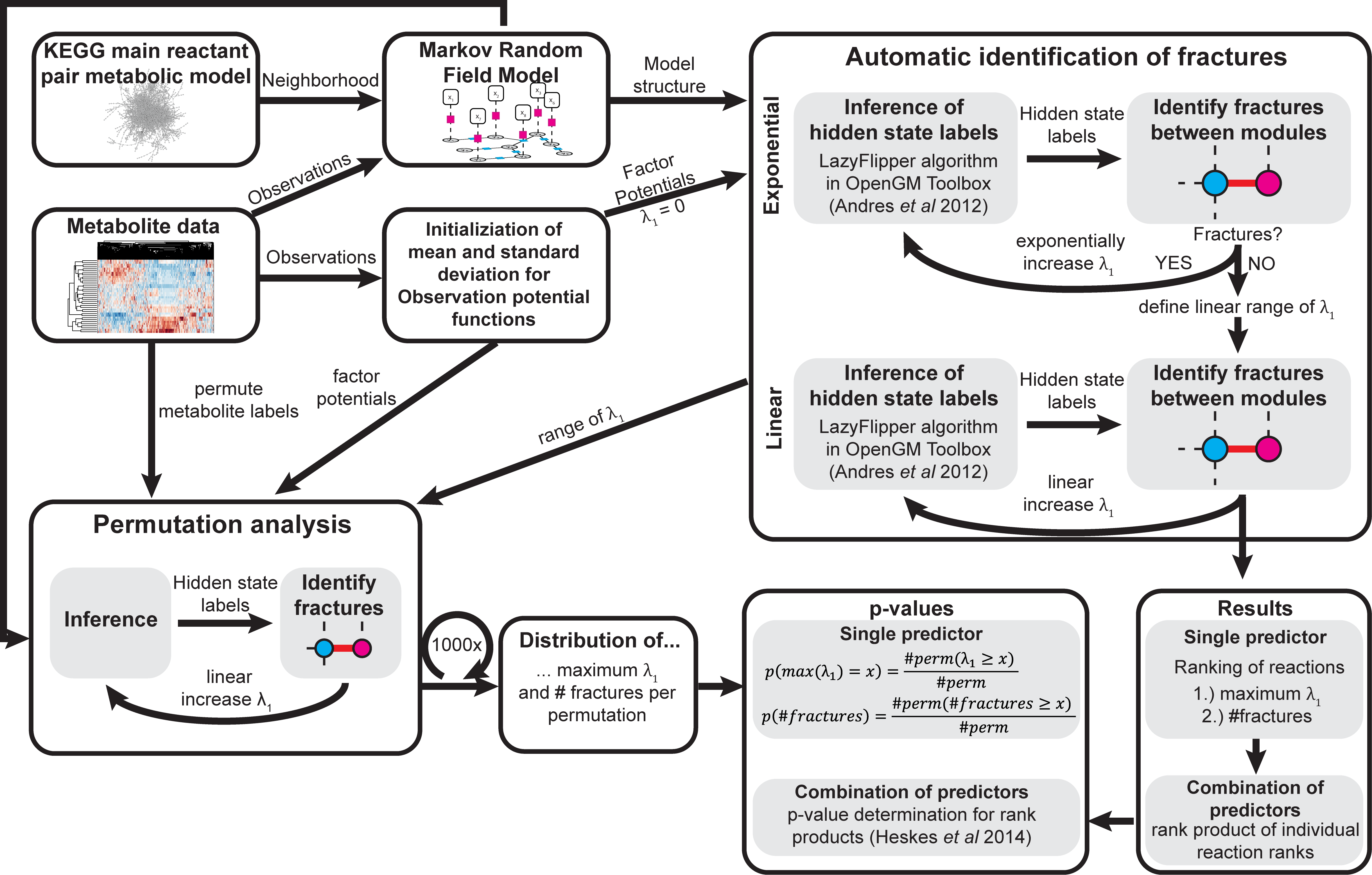

Supplement: S1 Fig — The algorithm integrates large-scale metabolomics data and metabolic models employing Markov random fields. The Markov random field model consist of a network of discrete hidden variables and of continuous observed variables for each detected metabolite. The (module) labels or states of the hidden variables describe to which module, i.e. a group of neighboring metabolites with similar metabolite changes, a certain metabolite belongs to. Observation potentials enforce a dependency between observations and the label of the hidden variables and neighborhood potentials enforce homogeneity of the module labels of neighboring metabolites, which is weighted by λ1. Given the metabolic observations and a certain model parameterization, the hidden state label distribution is inferred using the LazyFlipper algorithm from the OpenGM toolbox [25, 26]. The hidden state-label distribution is used to identify modules of similarly changing metabolites and the fractures between them, which we assume to represent sites of metabolic regulation. We infer the most relevant regulatory sites in a sequential scanning process with increasing influence of the neighborhood λ1. Since we assume that the most stable reactions represent biological meaningful regulations, we rank the reactions according to maximum λ1 at which a reaction is classified as fracture and according to the frequency of how often a reaction is classified as fracture. In case of combinations of multiple predictors with different parameterizations, the ranking is determined through rank products. Significance for a single predictor is assessed using a permutation test and for combination of predictors using significance test’s for rank products [28]. (TIF) [file pcbi.1005577.s001.tif]

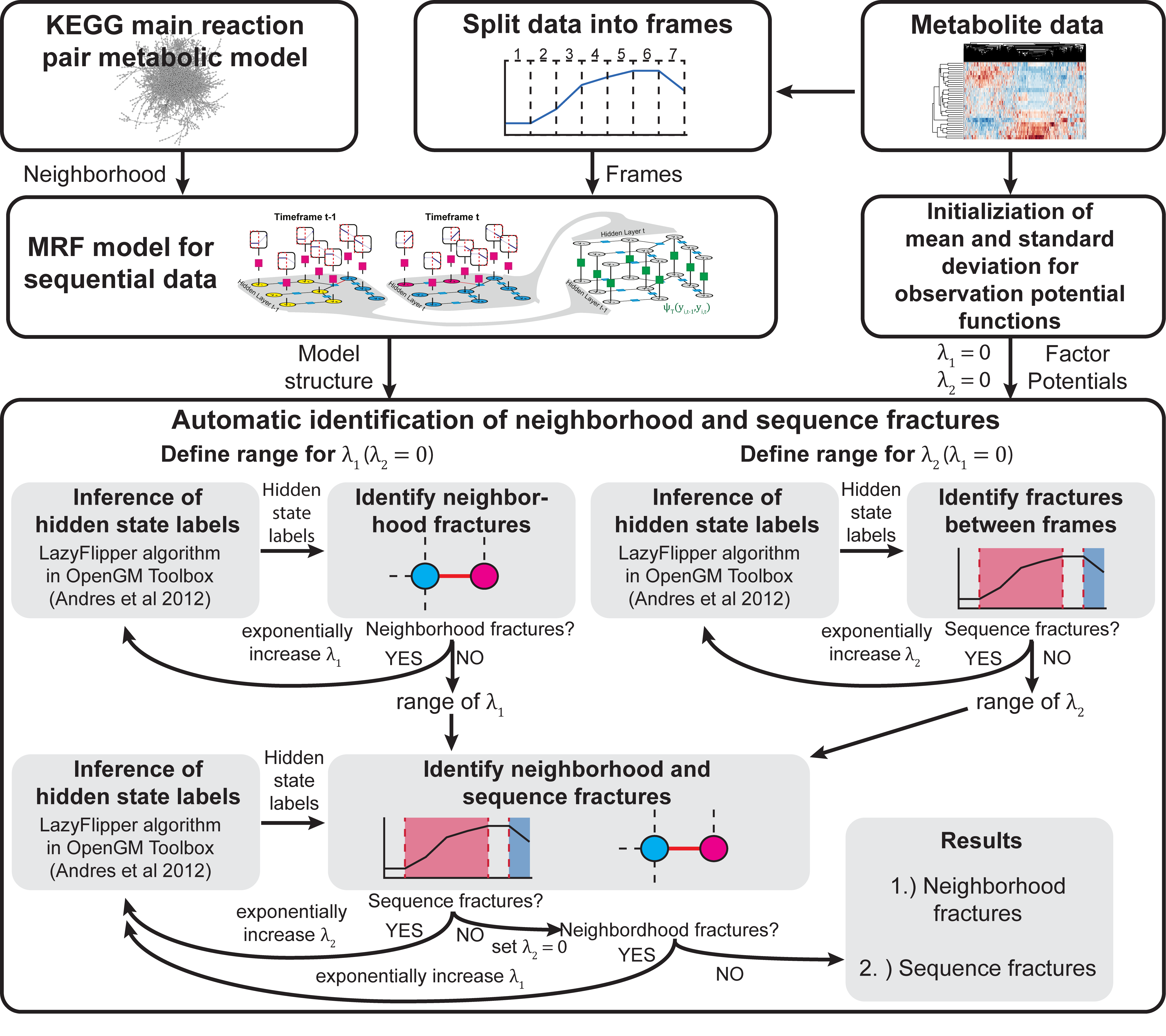

Supplement: S3 Fig — The algorithm splits sequential metabolomics data into individual frames and introduces for each frame a Markov random field model for univariate data (Fig 1b). The hidden states between neighboring sequential frames are connected by a sequence factor potential, which is weighted by λ1, to enforce a dependency between sequential data points. Similar to the MNS model for univariate data, we infer the most relevant regulatory sites and their sequential order in a step-wise scanning process with increasing influence of the neighborhood λ1 and sequential frames λ2 (S1 Fig). (TIF) [file pcbi.1005577.s003.tif]

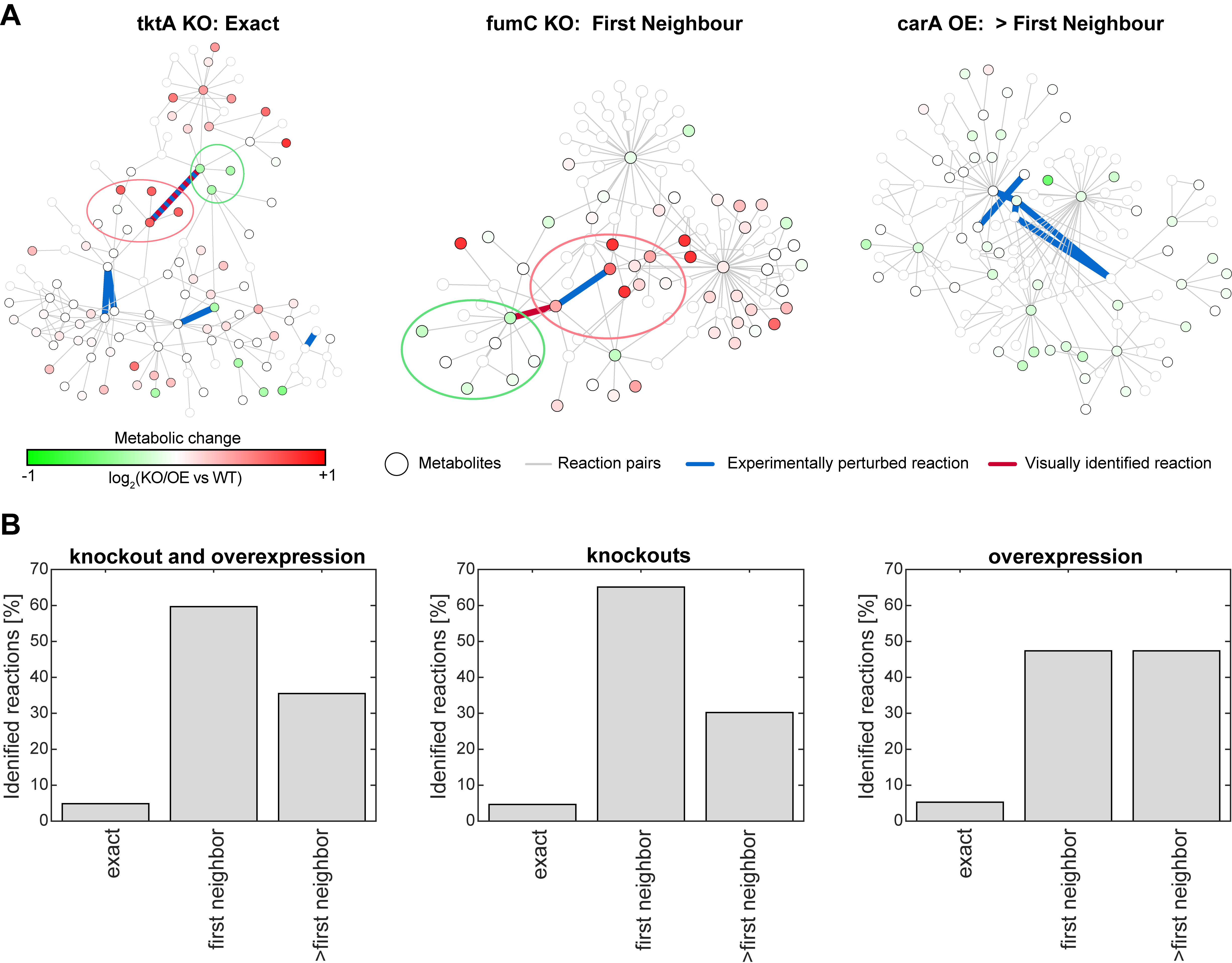

Supplement: S4 Fig — (a) Given the metabolite data mapped on a subnetwork around the perturbed reaction (maximal three reaction steps), two individual users inferred the perturbed reaction by visual inspection. Reactions are categorized into “exact” if the users identified the experimentally perturbed reaction (e.g. tktA KO), “first neighbor” if the users inferred one of the first neighbor reactions of the perturbed reaction (e.g. fumC KO) or “> first neighbor” if the inferred reaction was more than one reaction step away from the perturbed enzyme or was not identifiable at all (e.g. carA OE). (b) Only about 5% of the perturbed reactions were identified exactly, about 60% one of the first neighbor reactions was inferred, and more than 30% of the reactions were not identifiable. (TIF) [file pcbi.1005577.s004.tif]

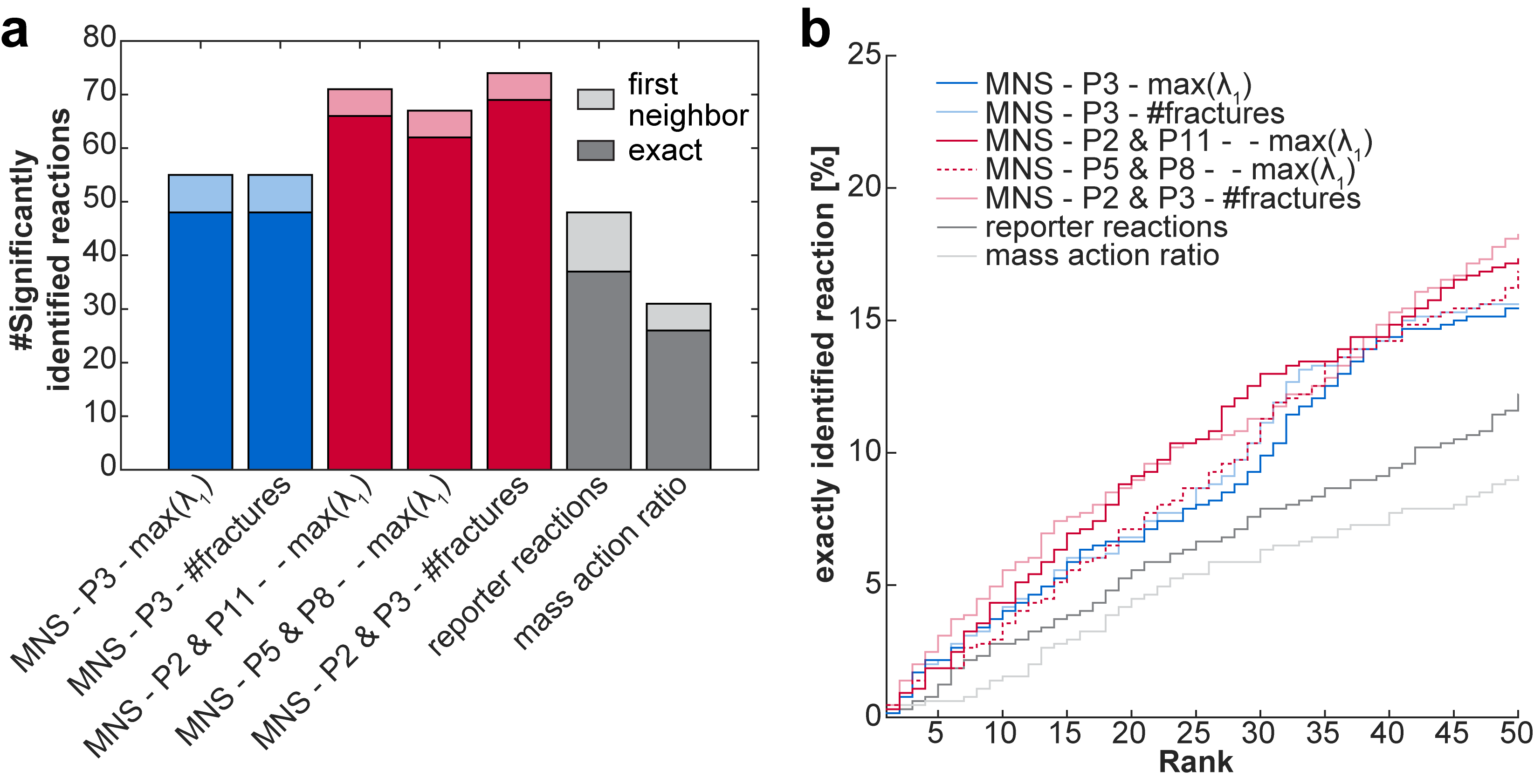

Supplement: S5 Fig — Independent of the parameterization the MNS algorithm outperforms current state-of-the-art methods in the identification of perturbed enzymes. (TIF) [file pcbi.1005577.s005.tif]

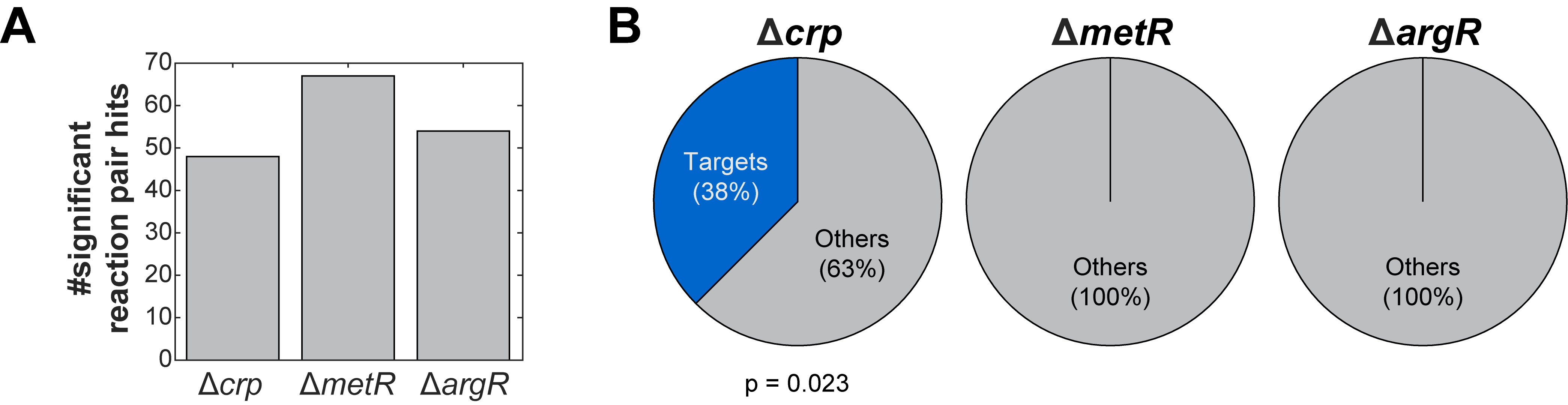

Supplement: S6 Fig — (a) Number of significantly identified reaction pairs (p < 0.01). Analysis was performed using a combination of the predictors with parameterization 2 and 3 (S1 Table). (b) Overlap between identified reaction pairs and reaction pairs that are known transcription factor targets. Ratio is determined comparing overlap and number of significantly predicted reaction pairs. p-value for Crp is calculated using a hypergeometric test. (TIF) [file pcbi.1005577.s006.tif]

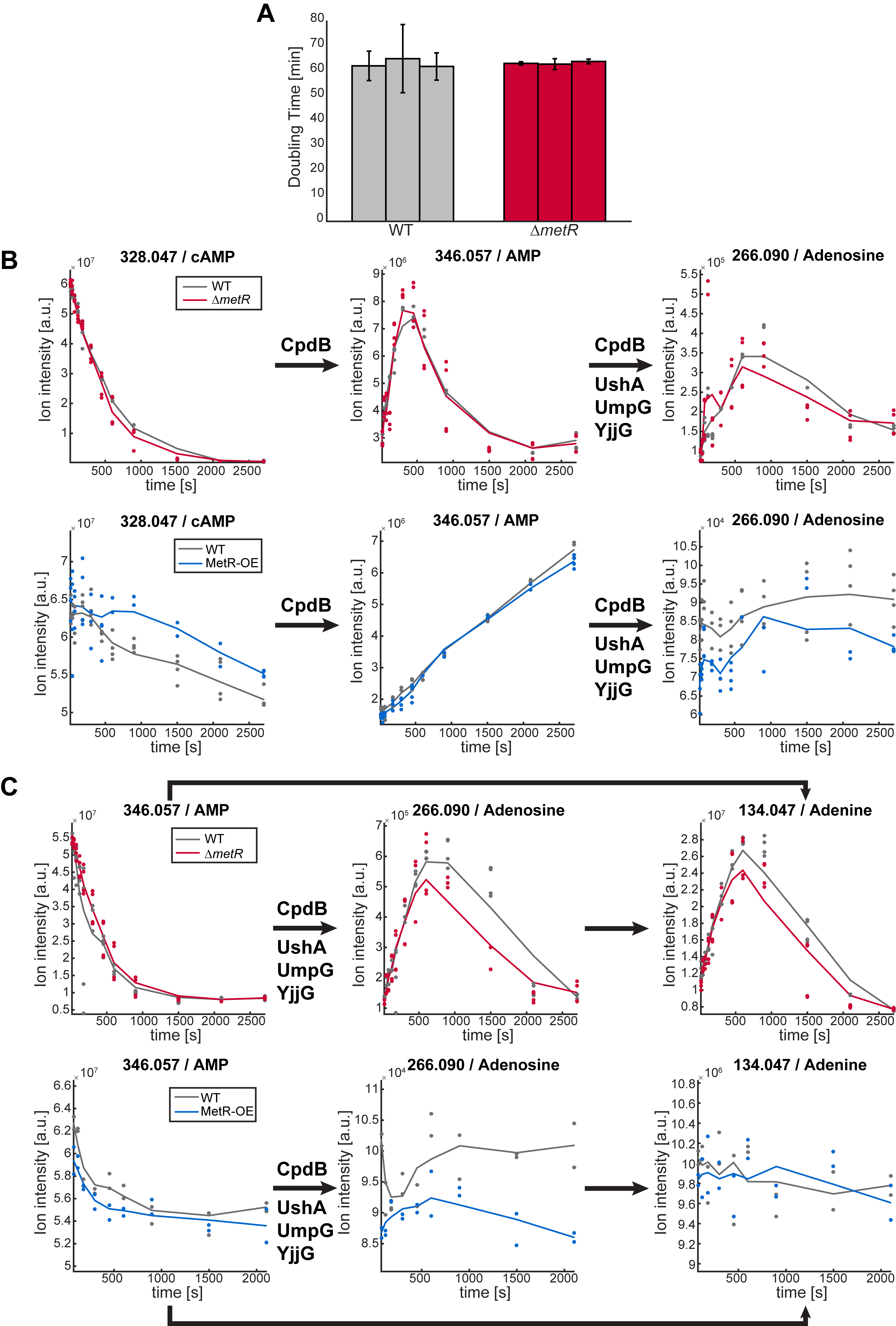

Supplement: S7 Fig — (a) Each bar represents mean values and standard deviation of doubling times at exponential growth phase of 4 individual biological replicates. There is no difference in doubling time comparing wild type E. coli and ΔmetR knockout mutants. (b,c) Enzyme assays with (b) 10 mM cAMP and (c) 10 mM AMP as substrate. Data shown represents mean values of 2 biological (except overexpression assays in c) and 2 technical replicates. (TIF) [file pcbi.1005577.s007.tif]

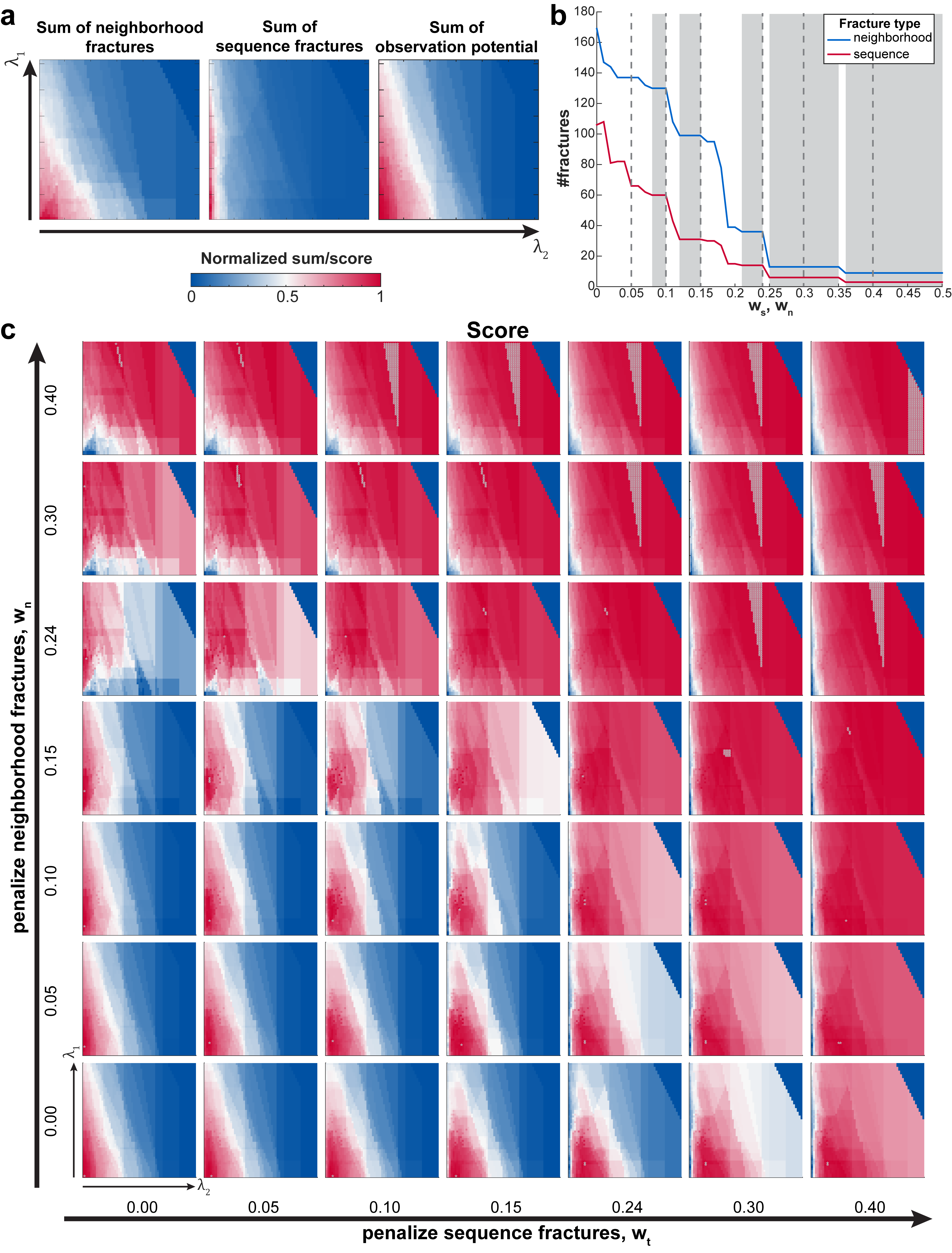

Supplement: S8 Fig — (a) Neighborhood and sequential influence λ1 and λ2 dependent distribution of sum neighborhood fractures, frame fracture and observation potential. (b) Total number of neighborhood and frame fracture with increasing penalization of fractures ws and wn. Shaded areas indicate pseudo-steady states of the fracture frequency, i.e. the number of fractures is not increasing for a certain range of weights. These are can be considered as module label distributions with a certain stability. Dashed lines indicate the weight values used for further analysis. (c) Influence of increasing penalization of sequence and neighborhood fractures on score (score = Sum of observation factor potential—ws*sum of sequence fractures—wn*sum of neighborhood fractures). White stars in (c) indicate the maximal score for the setting of ws an wn. With increasing penalization of fractures the optimal balance between number of fractures and the summed observation factor potential, i.e. how well do the model and the hidden module labels describe the data, can be determined. Score is normalized to a range between 0 and 1. (TIF) [file pcbi.1005577.s008.tif]

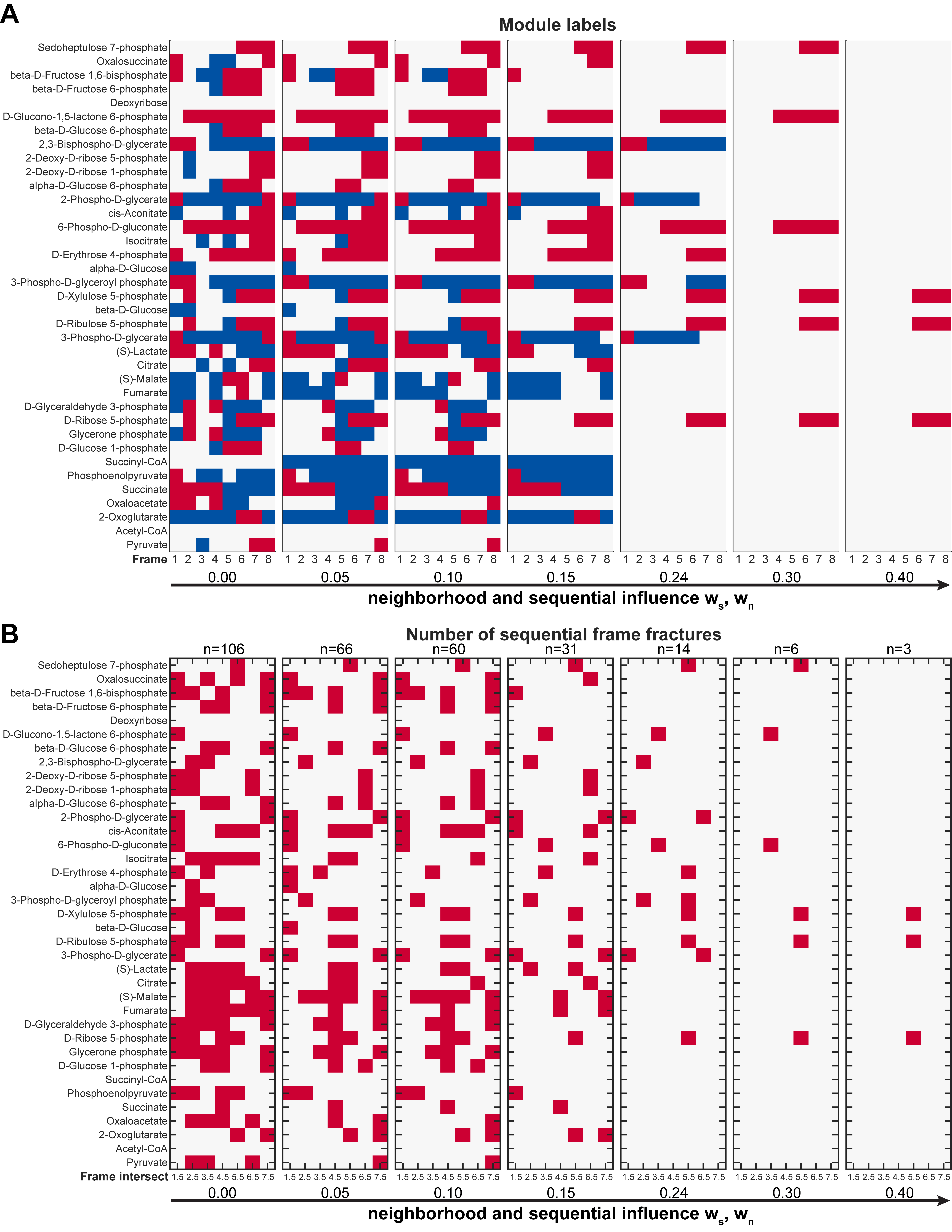

Supplement: S9 Fig — With increasing influence from metabolic neighborhood wn and sequential frames ws the (a) module label distribution gets homogenized and only the most important (b) sequence fractures remain. Weight values were determined in S8b Fig. (TIF) [file pcbi.1005577.s009.tif]

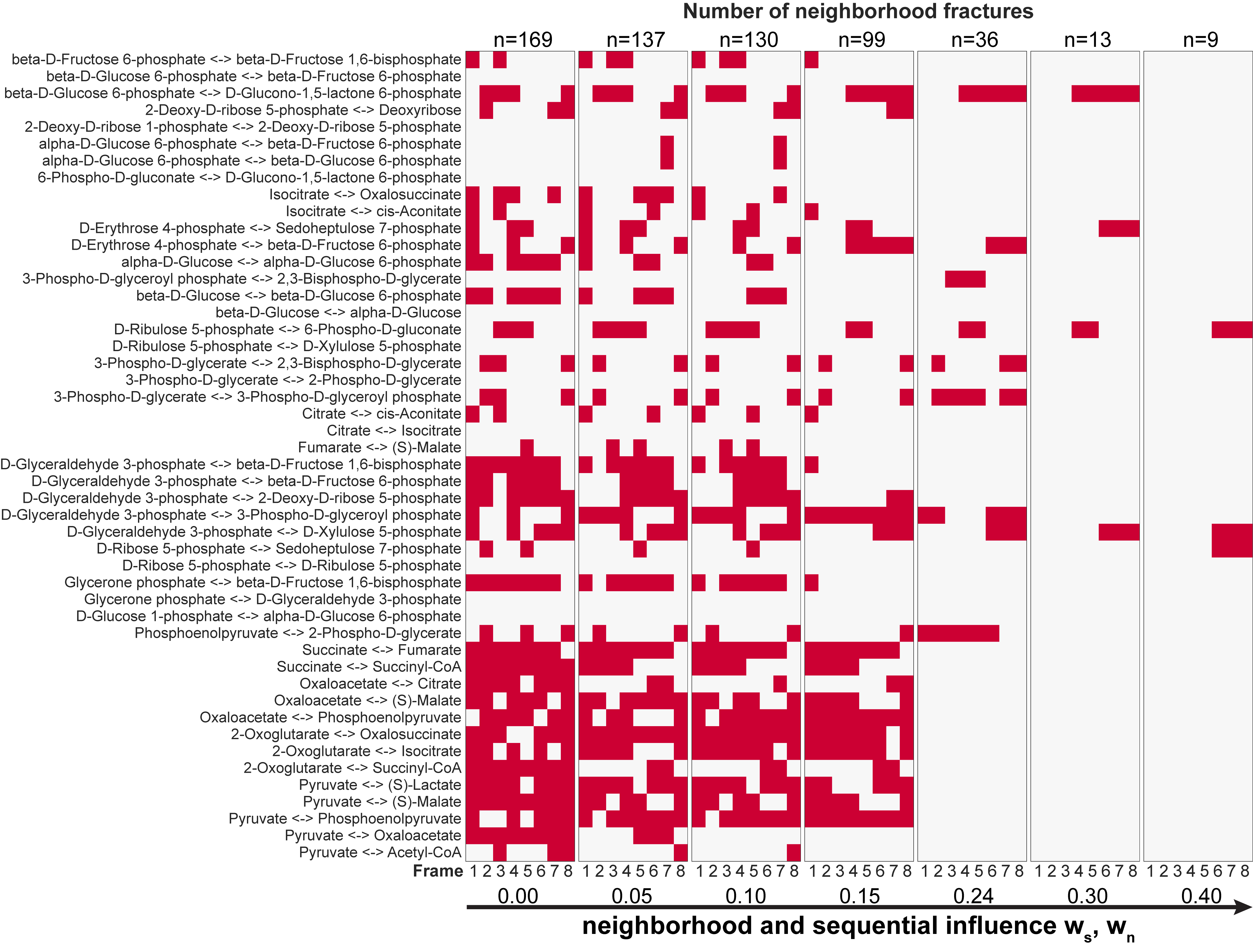

Supplement: S10 Fig — With increasing influence from metabolic neighborhood wn and sequential frames ws the neighborhood fracture distribution gets homogenized and only the most important fractures remain. Weight values were determined in S8b Fig. (TIF) [file pcbi.1005577.s010.tif]
