# Supplementary material for: Metabolic network segmentation: A probabilistic graphical modeling approach to identify the sites and sequential order of metabolic regulation from non-targeted metabolomics data
Source: PLoS Comput Biol. 2017 Jun 9;13(6):e1005577. doi: 10.1371/journal.pcbi.1005577 (PMC5482507; doi:10.1371/journal.pcbi.1005577)
Supplement: S1 Table — Description: No. of hidden states represents the number of different modules. Mean Type describes how the mean values of the observation potential function are derived (k-means: μ derived from the k-means clustering results; quantile: μ equally distributed between the 0.001 and 0.999 quantile of the data). Standard deviation type describes how the individual standard deviations of of the observation potential functions are determined (fix: fix standard deviation value for all hidden states σ = 1, k-means: individual σ for each hidden state equal to the standard deviation of the data points in a k-means cluster, all data: σ of all hidden states equal to standard deviation of the complete data). (PDF) [file pcbi.1005577.s011.pdf]

**Parameter Combination**	**No. of hidden states**	**Mean type**	**Standard deviation type**
1	3	k-means	fix
2			k-means
3			all data
4		quantile	fix
5			k-means
6			all data
7	4	k-means	fix
8			k-means
9			all data
10		quantile	fix
11			k-means
12			all data
13	5	k-means	fix
14			k-means
15			all data
16		quantile	fix
17			k-means
18			all data
