# Supplementary material for: Metabolic network segmentation: A probabilistic graphical modeling approach to identify the sites and sequential order of metabolic regulation from non-targeted metabolomics data
Source: PLoS Comput Biol. 2017 Jun 9;13(6):e1005577. doi: 10.1371/journal.pcbi.1005577 (PMC5482507; doi:10.1371/journal.pcbi.1005577)
Supplement: S2 Table — (PDF) [file pcbi.1005577.s012.pdf]

Type	Gene	Name	Pathway
knockout	*aceA*	Isocitrate lyase	Citric acid cycle
	aceB	Malate synthase (A)	Glycoxylate shunt
	acnB	2-methylisocitrate dehydratase	Citric acid cycle
	adhP	Alcohol dehydrogenase	Glycolysis
	aldA	Aldehyde dehydrogenase	Pyruvate metabolism
	argB	Acetylglutamate kinase	Arginine biosynthesis
	aroD	3-dehydroquinate dehydratase	Phenylalanine, tyrosine and tryptophan biosynthesis
	dadA	D-amino acid dehydrogenase	Alanine degradation
	edd	6-phosphogluconate dehydratase	Entner-Doudoroff-Pathway
	fbp	Fructose-1,6-bisphosphatase I	Glycolysis
	folP	7,8-dihydropteroate synthase	Folate biosynthesis
	fumA	Fumarase A (Isoenzyme I)	Citric acid cycle
	fumB	Fumarase B (Isoenzyme II)	Citric acid cycle
	fumC	Fumarase C (Isoenzyme III)	Citric acid cycle
	gcvP	Glycine decarboxylase	Glycine Cleavage System
	glyA	Serine hydroxymethyltransferase	Glycine, serine and threonine metabolism
	gnd	Phosphogluconate dehydrogenase	Pentose phosphate pathway
	gshA	Glutathione synthetase	glutathione biosynthesis
	gshB	Glutathione synthetase	glutathione biosynthesis
	ilvE	Branched-chain amino-acid aminotransferase	Valine, leucine and isoleucine metabolism
	ilvN	Acetolactate synthase 1 small subunit	Valine, leucine and isoleucine metabolism
	leuA	2-isopropylmalate synthase	Leucine synthesis
	maeB	Malic enzyme (Malat dehydrogenase NADP-dependent)	Pyruvate metabolism
	manA	Mannose-6-phosphate isomerase	Fructose and mannose metabolism
	mdh	Malate dehydrogenase	Citric acid cycle
	pabB	Aminodeoxychorismate lyase	Chorisimat Metabolism
	pabC	4-amino-4-deoxychorismate lyase	Folate biosynthesis
	pgi	Glucosephosphate isomerase	Glycolysis
	pgl	6-Phosphoglucono lactonase	Pentose phosphate pathway
	pgm	Phosphogluco mutase	Glycolysis
	ppc	Phosphoenolpyruvate carboxylase	Gluconeogenesis
	purM	Phosphoribosylaminoimidazole synthetase	Purine biosynthesis
	purN	5-aminoimidazole ribonucleotide biosynthesis I	Purine biosynthesis
	putA	Proline dehydrogenase	Proline degradation
	sdhB	Succinate dehydrogenase B	Citric acid cycle
	sdhC	Succinate dehydrogenase C	Citric acid cycle
	serB	Phosphoserine phosphatase	Serine Biosynthesis
	talA	Transaldolase A	Pentose phosphate pathway
	talB	Transaldolase B	Pentose phosphate pathway
	tktA	Transketolase II	Pentose phosphate pathway
	tpiA	Triosephosphate isomerase A	Glycolysis
	zwf	Glucose-6-phosphate dehydrogenase (oxidative)	Pentose phosphate pathway
overexpression	*accC*	Acetyl-CoA carboxylase (Biotin Carboxylase)	Fatty acid biosynthesis
	accD	Acetyl-CoA carboxylase (beta subunit)	Fatty acid biosynthesis
	argG	Argininosuccinate synthase	Urea cycle
	aroH	2-dehydro-3-deoxyphosphoheptonate aldolase	Shikimate biosynthesis
	carA	Carbomyl phosphate synthetase I (small subunit)	Urea cycle
	carB	Carbomyl phosphate synthetase I (large subunit)	Urea cycle
	fbp	fructose 1,6-bisphosphatase	Gluconeogenesis
	glpK	Glycerol kinase	Glycerolipid metabolism
	icd	Isocitrate dehydrogenase	Citric acid cycle
	metK	S-adenosylmethionine synthase	Cysteine and methionine metabolism
	pfkA	6-phosphofructokinase Isoenzyme PFK I	Glycolysis
	pfkB	6-phosphofructokinase Isoenzyme PFK II	Glycolysis
	purF	Glutamine-PRPP amidotransferase	Purine biosynthesis
	purL	phosphoribosylformyl-glycineamide synthetase	Purine biosynthesis
	pyrB	Aspartate carbamoyltransferase	Pyrimidine biosynthesis
	pyrK	Carbomyl phosphate synthetase II/Aspartate transcarbamoylase (Catalytic subunit)	Pyrimidine biosynthesis
	sucB	Dihydrolipoyllysine-residue succinyltransferase	Citric acid cycle
	thyA	Thymidylate synthase	Pyrimidine biosynthesis
	tyrA	Chorismate mutase	Tyrosine biosynthesis
	zwf	Glucose-6-phosphate dehydrogenase	Pentose phosphate pathway
