# Supplementary material for: Metabolic network segmentation: A probabilistic graphical modeling approach to identify the sites and sequential order of metabolic regulation from non-targeted metabolomics data
Source: PLoS Comput Biol. 2017 Jun 9;13(6):e1005577. doi: 10.1371/journal.pcbi.1005577 (PMC5482507; doi:10.1371/journal.pcbi.1005577)
Supplement: S3 Table — Significantly identified reactions were determined by a permutation test of the reaction labels with 1000 permutations and a p-value cutoff of 0.05. (PDF) [file pcbi.1005577.s013.pdf]

Parameter combination	Genes found in TOP10 Ranks [%]		#Significantly identified reactions		Significantly identified reactions [%]	
	Exact	Total	Exact	Total	Exact	Total
P1 - max(λ_1)	4.8	51.6	5	7	8.1	11.3
P1 - #fractures	4.8	53.2	5	8	8.1	12.9
P2 - max(λ1)	11.3	48.4	8	9	12.9	14.5
P2 - #fractures	9.7	53.2	8	10	12.9	16.1
P3 - max(λ1)	9.7	53.2	10	11	16.1	17.7
P3 - #fractures	9.7	54.8	11	13	17.7	21.0
P4 - max(λ1)	6.5	51.6	7	9	11.3	14.5
P4 - #fractures	4.8	48.4	7	10	11.3	16.1
P5 - max(λ1)	11.3	50.0	9	10	14.5	16.1
P5 - #fractures	9.7	48.4	7	9	11.3	14.5
P6 - max(λ1)	6.5	43.5	7	8	11.3	12.9
P6 - #fractures	4.8	41.9	7	9	11.3	14.5
P7 - max(λ1)	4.8	54.8	4	6	6.5	9.7
P7 - #fractures	6.5	59.7	4	6	6.5	9.7
P8 - max(λ1)	6.5	53.2	9	10	14.5	16.1
P8 - #fractures	4.8	53.2	6	8	9.7	12.9
P9 - max(λ1)	8.1	58.1	5	7	8.1	11.3
P9 - #fractures	6.5	61.3	6	8	9.7	12.9
P10 - max(λ1)	6.5	48.4	5	7	8.1	11.3
P10 - #fractures	8.1	56.5	6	8	9.7	12.9
P11 - max(λ1)	8.1	38.7	8	9	12.9	14.5
P11 - #fractures	4.8	37.1	6	8	9.7	12.9
P12 - max(λ1)	8.1	46.8	6	8	9.7	12.9
P12 - #fractures	6.5	50.0	7	9	11.3	14.5
P13 - max(λ1)	6.5	54.8	5	7	8.1	11.3
P13 - #fractures	6.5	54.8	5	7	8.1	11.3
P14 - max(λ1)	6.5	38.7	6	7	9.7	11.3
P14 - #fractures	4.8	40.3	5	6	8.1	9.7
P15 - max(λ1)	8.1	51.6	5	6	8.1	9.7
P15 - #fractures	6.5	53.2	6	8	9.7	12.9
P16 - max(λ1)	8.1	53.2	7	8	11.3	12.9
P16 - #fractures	8.1	58.1	7	9	11.3	14.5
P17 - max(λ1)	3.2	17.7	7	7	11.3	11.3
P17 - #fractures	4.8	43.5	9	9	14.5	14.5
P18 - max(λ1)	6.5	38.7	8	8	12.9	12.9
P18 - #fractures	4.8	46.8	7	7	11.3	11.3
P2&P11 - max(λ1)	6.5	54.8	14	14	22.6	22.6
P2&P11 - #fractures	6.5	58.1	12	13	19.4	21.0
P5&P8 - max(λ1)	9.7	51.6	13	14	21.0	22.6
P5&P8 - #fractures	8.1	53.2	10	13	16.1	21.0
P2&P3 - max(λ1)	11.3	58.1	11	12	17.7	19.4
P2&P3 - #fractures	9.7	58.1	12	14	19.4	22.6
