# Supplementary material for: Metabolic network segmentation: A probabilistic graphical modeling approach to identify the sites and sequential order of metabolic regulation from non-targeted metabolomics data
Source: PLoS Comput Biol. 2017 Jun 9;13(6):e1005577. doi: 10.1371/journal.pcbi.1005577 (PMC5482507; doi:10.1371/journal.pcbi.1005577)
Supplement: S4 Table — The analysis was performed with a combination of two parameterizations (S1 Table, P2 & P3). (PDF) [file pcbi.1005577.s014.pdf]

Rank	RP ID	EC	Gene Symbol	Reaction	rankproduct (#fractures)	p-value
1	RP02160	3.1.3.6	*cpdB*	Cytidine ↔ 3'-CMP	8	7.24E-06
3	RP01744	3.2.2.8	*rihB*	D-Ribose ↔ Inosine	22	2.74E-05
3	RP00466	2.7.1.73; 3.1.3.5	*gsk; ushA; umpG; yjjG*	IMP ↔ Inosine	22	2.74E-05
3	RP00175	2.4.2.1; 3.2.2.8	*deoD; rihB*	Hypoxanthine ↔ Inosine	22	2.74E-05
5	RP00895	4.6.1.1	*cyaA*	GTP ↔ 3',5'-Cyclic GMP	32	4.39E-05
6	RP03430	4.6.1.1	*cyaA*	ATP ↔ 3',5'-Cyclic AMP	48	7.26E-05
7.5	RP00453	3.2.1.28	*treA; treF*	D-Glucose ↔ Trehalose	60	9.53E-05
7.5	RP00343	2.7.1.69; 3.1.3.12	*yadI; nagE; …*	Trehalose 6-phosphate ↔ Trehalose	60	9.53E-05
9	RP04731	3.2.1.86	*chbF; ascB; bglA; bglB*	Salicin 6-phosphate ↔ Salicyl alcohol	72	0.000119
10	RP02000	3.1.3.6	*cpdB*	Guanosine ↔ Guanosine 3'-phosphate	161.25	0.000312
11	RP01596	3.1.3.6	*cpdB*	Adenosine ↔ 3'-AMP	180	0.000355
