# Supplementary material for: Metabolic network segmentation: A probabilistic graphical modeling approach to identify the sites and sequential order of metabolic regulation from non-targeted metabolomics data
Source: PLoS Comput Biol. 2017 Jun 9;13(6):e1005577. doi: 10.1371/journal.pcbi.1005577 (PMC5482507; doi:10.1371/journal.pcbi.1005577)
Supplement: S1 Text — (PDF) [file pcbi.1005577.s015.pdf]

Details on the Parameter optimization

To optimize the prediction of regulatory sites by the MNS algorithm, we tuned first the three most influential parameters of our algorithm: number of hidden states, determination mode of mean values for the observation potential functions and determination mode of standard deviation for the observation potential functions (S1 Table). As training dataset with known regulatory sites we analyzed the metabolite changes of 62 *Escherichia coli* single enzyme knockouts and overexpression mutants using non-targeted metabolomics analysis (S2 Table). For most parameter combinations, our algorithm identified between 5 to 11% of the perturbed enzymes exactly and in 40 to 60% of the cases one of the first neighbor reactions of the perturbed enzyme within the ten highest ranked predictions (S3 Table). This regulatory site inference achieved with the MNS algorithm is comparable to the manual identification of the perturbed enzymes by an expert scientist (S4 Fig). Furthermore, to evaluate whether the algorithm predicts the perturbed enzyme significantly better than a random guess we performed a permutation test of the reaction labels. The test demonstrated that while for most parameter combinations between 7 and 10 perturbed reactions were significantly identified ($p < 0.05$, permutation test), for one outstanding parameter combination P3 (3 hidden state labels, k-means dependent mean values, whole data dependent standard deviation) 11 (maximum λ_1) and 13 (fracture frequency) of the perturbed enzymes were predicted significantly (Fig 3a, S7 Table).

Interestingly, a comparison of the performance of our approach given the individual parameter combinations indicated that for some enzymatic perturbations, for example *sdhC* knockout, a different parameter set would be preferable (Fig 3b). Inspired by the notion that a combination of multiple predictors can improve the prediction results [21-24], we tested the performance of pairwise combinations of independent predictions obtained with different parameters integrated by their rank product. Three individual combinations (P2/P11/max(λ_1), P5/P8/max(λ_1), P2/P3/#fractures) of the parameter sets slightly improved the prediction results to 14 (22.6%) significantly identified reactions ($p < 0.05$, permutation test; Fig 3c, S3 Table).
