# Supplementary material for: Metabolic network segmentation: A probabilistic graphical modeling approach to identify the sites and sequential order of metabolic regulation from non-targeted metabolomics data
Source: PLoS Comput Biol. 2017 Jun 9;13(6):e1005577. doi: 10.1371/journal.pcbi.1005577 (PMC5482507; doi:10.1371/journal.pcbi.1005577)
Supplement: S2 Text — (PDF) [file pcbi.1005577.s016.pdf]

Details on the determination of λ_1 and λ_2 scanning ranges for the application of the MNS model for sequential data on hydrogen peroxide treated fibroblasts.

To illustrate the potential of our MNS algorithm to predict sites and sequential order of metabolic regulations given sequential metabolomics data, we applied our algorithm on a previously published metabolomics dataset from fibroblasts treated for 5 minutes with 9 different H_2O_2 concentrations ranging from 0 – 500 µM [1]. We ran the algorithm with three clusters with fix mean values ($\mu_1 = -0.1, \mu_2 = 0, \mu_3 = 0.1$), data dependent standard deviation values for the observation function. Since the inference of sites and sequential order of regulations is very time consuming we first performed a coarse grained search through the parameters in which the parameter ranges of λ_1 and λ_2 are defined automatically as described in the method section. The analysis indicated that with increasing influence of the neighborhood and sequence weights w_n and w_s, the number of fractures decrease (Fig 1, left). While for w_s and w_t = 0.21 the algorithm identifies dozens of sequence and neighborhood fractures, for w_s and w_t > 0.21 all metabolites are in the same module except 2,3-bisphosphoglycerate, so that there are only one sequential and two neighborhood fractures left (Fig 2). Since those inferred fractures are not biological meaningful, we used $w_s = w_n$ = 0.21 as reference point and investigated the fracture stability in a more fine grained scan through λ_1 and λ_2 in the range from 0 to 0.6, which represents approximately double the maximal λ values of $w_s = w_n$ = 0.21 ($\lambda_1(w_s = w_n = 0.21) = 0.29$ and $\lambda_2(w_s = w_n = 0.21) = 0.18$).

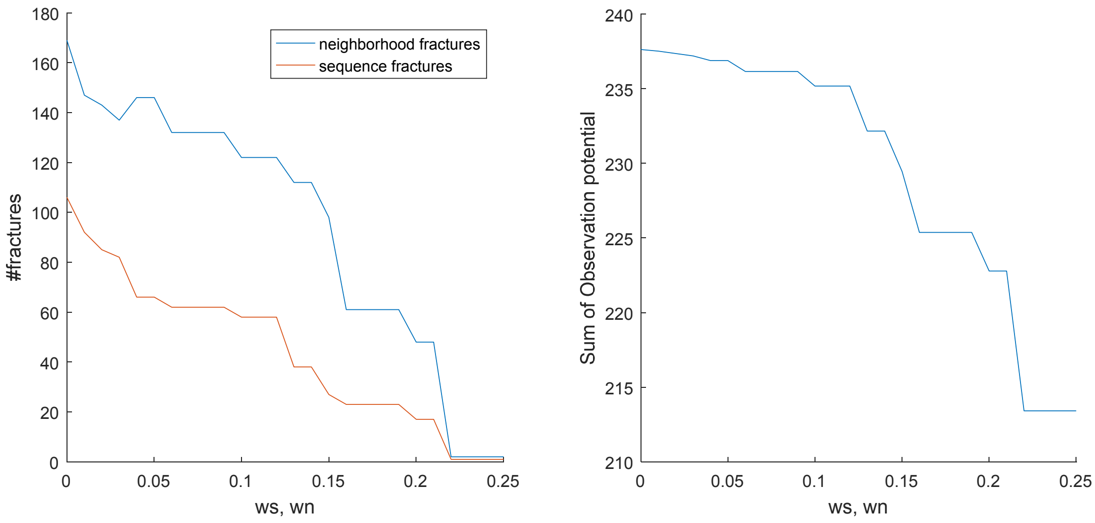

Fig 1 Total number of neighborhood and sequence fractures (left) and sum of observation potential (right) for increasing neighborhood and sequence weights w_n and w_s.

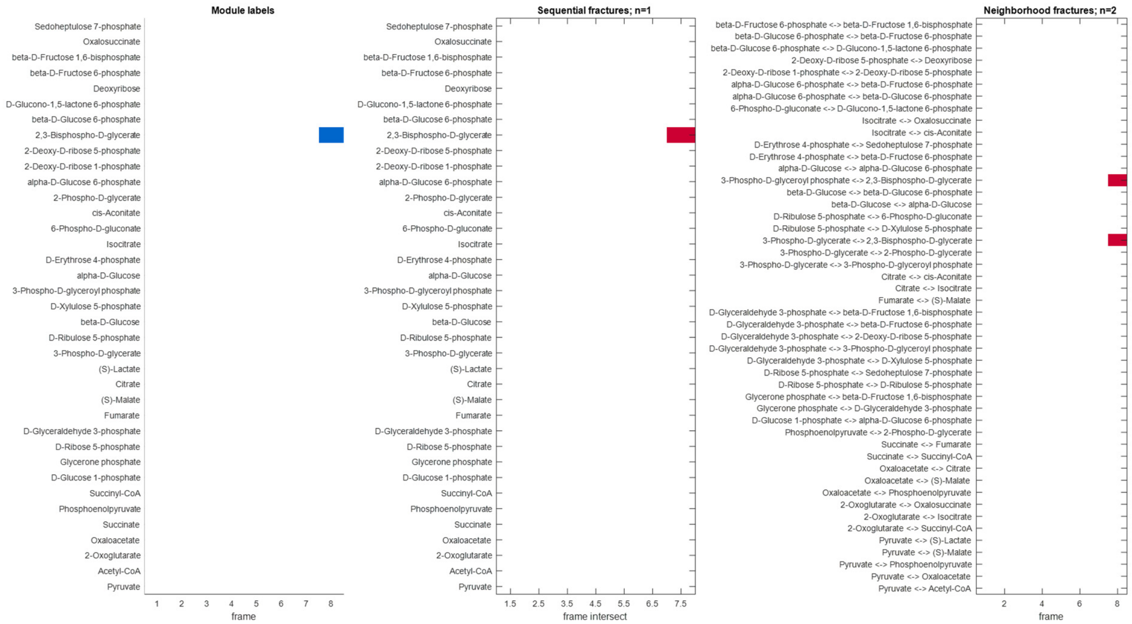

Fig 2 Overview of module labels (left), sequence fractures (middle) and neighborhood fractures (right) for increasing neighborhood and sequence weights w_n and w_s.

1. Kuehne A, Emmert H, Soehle J, Winnefeld M, Fischer F, Wenck H, et al. Acute Activation of Oxidative Pentose Phosphate Pathway as First-Line Response to Oxidative Stress in Human Skin Cells. Mol Cell. 2015;59(3):359-71. doi: 10.1016/j.molcel.2015.06.017.
